# *De Novo* Sequencing Provides Insights Into the Pathogenicity of Foodborne *Vibrio parahaemolyticus*

**DOI:** 10.3389/fcimb.2021.652957

**Published:** 2021-05-14

**Authors:** Jianfei Liu, Kewei Qin, Chenglin Wu, Kaifei Fu, Xiaojie Yu, Lijun Zhou

**Affiliations:** ^1^ Central Laboratory, The Sixth Medical Centre, Chinese PLA (People’s Liberation Army) General Hospital, Beijing, China; ^2^ College of Otolaryngology Head and Neck Surgery, The Sixth Medical Centre, Chinese PLA (People’s Liberation Army) General Hospital, Beijing, China

**Keywords:** *Vibrio parahaemolyticus*, pathogenicity, *de novo* sequencing, virulence, mouse model

## Abstract

*Vibrio parahaemolyticus* is a common pathogenic marine bacterium that causes gastrointestinal infections and other health complications, which could be life-threatening to immunocompromised patients. For the past two decades, the pathogenicity of environmental *V. parahaemolyticus* has increased greatly, and the genomic change behind this phenomenon still needs an in-depth exploration. To investigate the difference in pathogenicity at the genomic level, three strains with different hemolysin expression and biofilm formation capacity were screened out of 69 environmental *V. parahaemolyticus* strains. Subsequently, 16S rDNA analysis, *de novo* sequencing, pathogenicity test, and antibiotic resistance assays were performed. Comparative genome-scale interpretation showed that various functional region differences in pathogenicity of the selected *V. parahaemolyticus* strains were due to dissimilarities in the distribution of key genetic elements and in the secretory system compositions. Furthermore, the genomic analysis-based hypothesis of distinct pathogenic effects was verified by the survival rate of mouse models infected with different *V. parahaemolyticus* strains. Antibiotic resistance results also presented the multi-directional evolutionary potential in environmental *V. parahaemolyticus*, in agreement with the phylogenetic analysis results. Our study provides a theoretical basis for better understanding of the increasing pathogenicity of environmental *V. parahaemolyticus* at the genome level. Further, it has a key referential value for the exploration of pathogenicity and prevention of environmental *V. parahaemolyticus* in the future.

## Introduction

*Vibrio parahaemolyticus* is a gram-negative halophilic bacterial species, first identified in 1950 (Fujino et al., 1953). Various serotypes of *V. parahaemolyticus* have been confirmed to be opportunistically pathogenic to humans (Ueno et al., 2016), causing acute or subacute gastroenteritis accompanied with dehydration, chills, and fever, irrespective of age or gender (Yang et al., 2019b). Primary symptoms can easily progress to severe dehydration, causing shock and other concomitant complications, which can even result in the death of immunocompromised patients, if not treated in a timely manner (Shuja et al., 2014; Guillod et al., 2019). Due to its wide existence in marine and terrestrial environments (Miyamoto et al., 1962; Barker, 1974), *V. parahaemolyticus* causes foodborne diseases worldwide. It is the only marine bacterium among the top five foodborne pathogens causing human infection (CDC, 2019). Although this bacterium was normally known to cause infections locally, since the past two decades, there have been reports of transcontinental distribution of certain *V. parahaemolyticus* strains (Miyamoto et al., 1962; Yang et al., 2019a). There has been an increase in the number of clinical cases of *V. parahaemolyticus* infection (Li et al., 2016), and sporadic or epidemic cases have been reported in some regions that were once considered unsuitable for the growth of *V. parahaemolyticus*, such as South America and Northern Europe (Martinez-Urtaza et al., 2010; Baker-Austin et al., 2013). This suggests that *V. parahaemolyticus* is continuously evolving and is developing into a more serious pathogen (Nair et al., 2007; Newton et al., 2014). However, the mechanisms of its rapid serotype conversion and pandemic evolution have not been revealed yet.

Recently, pathogenic feature changes of environmental *V. parahaemolyticus* strains suggested a high level of genetic diversity and a more rapid recombination frequency, strongly connected with highly complex and fast genomic evolution (Yang et al., 2019b), which are worthy of in-depth exploration. However, mainstream research methods, such as serotyping, multilocus sequence typing, and pulsed field gel electrophoresis, have been unable to fully explain the evolutionary attributes of environmental *V. parahaemolyticus* and the cause of its enhanced pathogenicity. Whole Genome Sequencing (WGS) technology is an emerging molecular researching method (Schürch et al., 2018), which provides a new way to study microorganisms through correlation analysis (Rossen et al., 2018), for resolving and reconstructing sample sequences and predicting detailed genomic functions by comparison to public databases. The convenience of genomic analysis by WGS technology makes it a powerful method for understanding genome properties more deeply, tracing back the phylogenetic progress, and predicting the pathogenicity of microorganisms (Ronholm et al., 2016; Gonzalez-Escalona et al., 2017). Therefore, a study on *V. parahaemolyticus* pathogenicity by WGS could provide a more comprehensive interpretation of the rapid evolutionary changes occurring in this species.

In this paper, virulence features of 69 V*. parahaemolyticus* strains derived from food products were evaluated by analyzing the hemolysin-encoding gene and differences in biofilm formation (BF), and three strains were selected for the further study. In order to interpret the difference in pathogenicity at the genomic level, *de novo* sequencing was used to understand the genetic composition and to explore the functional information. Pathogenicity *in vivo* and antibiotic resistance were also evaluated. Our study provided a more comprehensive idea for developing a better understanding of foodborne *V. parahaemolyticus* and for further interpreting the relationship between the pathogenicity and genomic changes of environmental *V. parahaemolyticus*.

## Materials and Methods

### Bacteria and Growth Conditions

A total of 69 environmental strains of *V. parahaemolyticus* were used in this study (hereafter referred to as strains *Vp.* 1463–1528, *Vp.* 4213, *Vp.* 4215, and *Vp.* 11577), which were kindly provided as a gift by Prof. Shenghui Cui, who had maintained them at the National Institutes for Food and Drug Control (NIFDC) of China. Original *V. parahaemolyticus* were isolated from commercial food samples during the day of August 25, 2015, to June 1, 2016, and were land transported from Anhui, Shanxi and Fujian provinces in China, respectively. Detailed background information could be found in **Table S1**. All *V. parahaemolyticus* strains were initially maintained on 2216E agar plates (BD Biosciences, NJ, USA) at 35°C for 12 h, and routinely cultured in 2216E broth (BD Biosciences, NJ, USA) at 35°C, with shaking at 180 rpm for 12 h. Dilution or enrichment of cultured *V. parahaemolyticus* strains was performed in 3% NaCl alkaline peptone water (APW; Land Bridge Technology, Beijing, China).

### Multiplex PCR Assay for *V. parahaemolyticus* Hemolysins

The culture density of all 69 V*. parahaemolyticus* strains was adjusted to 3 × 10^5^ CFU/ml, and they were cultured in 3% NaCl APW at 35°C with shaking at 180 rpm. After culturing for 6 h, 1 ml of the cultures was centrifuged (10,000 rpm) to obtain a bacterial pellet from which genomic DNA was extracted using a DNA extraction kit (TIANGEN, Beijing, China). Concentrations of genomic DNA were measured using NanoDrop 8000 (Thermo Fisher, MA, USA).

Three pairs of primers targeting genes *tdh*, *trh*, and *tlh* were designed for PCR based on their DNA sequences (**Table S2**), using the Primer Premier 5.0 software (San Francisco, CA, USA; http://www.premierbiosoft.com/primerdesign/). Multiplex PCR amplification was optimized in a 50-μl reaction consisting of 0.5 mg purified genomic DNA, 1 μM of each primer, 25 μl of GoTaq qPCR Master Mix (Promega Corporation, WI, USA), and an appropriate volume of sterile water (Milli-Q; Merck, Darmstadt, Germany).

All multiplex PCR amplifications were performed on an ABI 2720 Thermal Cycler (Thermo Scientific, MA, USA), using the following temperature-cycling parameters: initial denaturation at 94°C for 3 min, followed by 30 cycles of amplification, with each cycle involving denaturation at 94°C for 1 min, primer annealing at 58°C for 1 min, and primer extension at 72°C for 1 min. After the amplification cycles, samples were kept at 72°C for 5 min to allow the final extension of the incompletely synthesized DNA.

### Biofilm Formation Assay

The semi-quantitative adhesion test was performed for assessing biofilm formation, using the method described by Stepanovic et al., with some modifications (Stepanovic et al., 2000). In brief, cultures of all 69 V*. parahaemolyticus* strains were adjusted to 3 × 10^5^ CFU/ml, and incubated in 96-well polystyrene microplates (Corning, NY, USA), at 35°C for 30 h. Suspended cultures were transferred to a fresh microplate to measure the CFU. The original microplate was first rinsed three times with phosphate buffered saline (PBS; Leagene, Beijing, China), followed by fixing with Bouin’s fluid (Leagene, Beijing, China) for 20 min. Then the microplate was rinsed three more times with PBS, and subsequently stained with crystal violet (Leagene, Beijing, China) for 30 min, followed by a final rinsing with PBS three times. Stained biofilm on each air-dried well was washed with crystal violet using ethanol (95%, v/v solution; Sinopharm, Beijing, China) until it was completely rinsed off. An iMark microplate reader (Bio-Rad, CA, USA) was used for measuring the absorbance at 570 nm (OD_570 nm_). The normalized BF was calculated using the following formula: normalized BF_sample_ = (original BF_sample_ − BF_control_)/log_(CFU/ml)_.

### Phylogenetic Analysis of *V. parahaemolyticus*

All *V. parahaemolyticus* strains were sequenced by 16S rDNA sequencing, and the general pair of primers were as follows: 27F (5′-AGAGTTTGATCCTGGCTCAG-3′) and 1492R (5′-TACGGCTACCTTGTTACGACTT-3′) (Lane, 1991). 16S rDNA sequences were assembled for phylogenetic analysis. To further determine the degree of molecular evolution, three strains, viz., *Vp.* 1474, *Vp.* 1496, and *Vp.* 1513, were selected based on the differences in the expression of hemolysin genes *tdh* and *trh*, and BF capacity. *V. probioticus* LMG 20362T, *V. rotiferianus* LMG 21460T, *V. proteolyticus* ATCC 15338T, *V. parahaemolyticus* ATCC 17802T, *V. natriegens* ATCC 14048T, *V. harveyi* NCIMB 1280T, *V. campbellii* ATCC 25920T, and *V. alginolyticus* ATCC 17749T were used as reference *Vibrio* strains for phylogenetic analysis (**Table S1**). Phylogenetic and molecular evolutionary analyses were conducted using MEGA version 6 (Pennsylvania State University, PA, USA; https://www.megasoftware.net/). The maximum likelihood tree was used for reconstructing the phylogenetic process, and detailed analyses, including phylogenetic tests on the nucleotides, were conducted using the Tamura-Nei substitution model based on a 1,000-replication bootstrap method.

### *De Novo* Sequencing

#### Extraction of Genomic DNA

The strains *Vp.* 1474, *Vp.* 1496, and *Vp.* 1513 were cultured overnight in 3% NaCl APW, and genomic DNA was extracted from them using a DNA extraction kit (TIANGEN, Beijing, China). The harvested DNA was detected using agarose gel electrophoresis, and quantified using Nanodrop 8000 (Thermo Scientific, MA, USA).

#### Library Construction and Sequencing

Total 1 μg DNA for each of the three strains was used as input material for the DNA sample preparations. Sequencing libraries were generated using NEBNext^®^ Ultra™ DNA Library Prep Kit for Illumina (New England BioLabs Inc., MA, USA), following the manufacturer’s recommendations. Briefly, the genomic DNA was fragmented by sonication to sizes of 350 bp and 6 kb. Large DNA fragments were amplified with circularization amplification. The products were randomly disrupted into fragments of about 350 bp, and the sequences on both sides of the circularized primers were captured with probes for subsequent DNA library construction. DNA fragments were end-polished, added poly-A tail, and ligated with the full-length adaptor for further PCR amplification. At last, PCR products were purified using AMPure XP system (Beckman Coulter, CA, USA), and the insert size of libraries was analyzed for size distribution by Agilent Bioanalyzer 2100 (Agilent, CA, USA), and the libraries were quantified using real-time PCR (Roche, Basel, Switzerland).

The genomes of *Vp.* 1474, *Vp.* 1496, and *Vp.* 1513 were separately sequenced using Illumina NovaSeq PE150 (Beijing Compass Bioinformatics Technology Co., Ltd., Beijing, China). Data analyses, including genome assembly analysis, genome component prediction, gene function prediction, and comparative genomics analysis, were conducted.

#### Bioinformatics Analysis

The GeneMarkS software (version 4.17; Georgia Institute of Technology, GA, USA; http://exon.gatech.edu/GeneMark/genemarks.cgi) was used to predict coding genes for the sequenced genomes of *Vp.* 1474, *Vp.* 1496, and *Vp.* 1513 (Besemer et al., 2001). Based on the sequence composition, the IslandPath-DIOMB software (version 0.2; Simon Fraser University, Vancouver, Canada; http://www.pathogenomics.sfu.ca/islandpath/) was used to predict genomic islands (GIs) (Bertelli and Brinkman, 2018). Determination of GIs by detecting phylogenetic bias and mobility genes in the sequence also enabled the detection of potential horizontal gene transfers (HGT). PhiSpy software (version 3.4; Stanford University, CA, USA; https://github.com/linsalrob/PhiSpy) was used to predict prophage loci on the genome of *Vp.* 1474, *Vp.* 1496 and *Vp.* 1513 (Akhter et al., 2019). CRISPRdigger software (version 1.0; Chinese Academy of Sciences, Shenzhen, China; https://github.com/greyspring/CRISPRdigger) was used for identifying CRISPR sequences in the genome of the three selected *V. parahaemolyticus* strains (Ge et al., 2016). Predicted gene-encoding protein sequences were aligned using the Diamond software (version 2.0.1; Max Planck Institute for Developmental Biology, Tübingen, Germany, http://www.diamondsearch.org), and the gene matching e-values ≤ 1^e-5^ were screened. Based on the alignment results of each sequence, the alignment with the highest score (default identity≥ 40%, coverage≥ 40%) was selected for annotation.

Three databases, viz., Gene Ontology (GO; http://geneontology.org/), Kyoto Encyclopedia of Genes and Genomes (KEGG; https://www.genome.jp/kegg/), and Clusters of Orthologous Groups (COG; http://clovr.org/docs/clusters-of-orthologous-groups-cogs/), were used for predicting gene functions of *Vp.* 1474, *Vp.* 1496, and *Vp.* 1513. For virulence and pathogenicity analysis, Pathogen-Host Interactions database (PHI; https://www.uniprot.org/database/phi-base), Antibiotic Resistance Genes Database (ARDB; http://ardb.cbcb.umd.edu/), Comprehensive Antibiotic Research Database (CARD; https://card.mcmaster.ca/), and Virulence Factors Database (VFDB; http://www.mgc.ac.cn/VFs/) were used. Subsequently, multiple analysis tools were used to predict the gene-encoding effectors, which included the secretory protein prediction with SignalP (version 4.1; Technical University of Denmark, Copenhagen, Denmark; http://www.cbs.dtu.dk/services/SignalP/) and TMHMM software (version 2.0c; Technical University of Denmark, Copenhagen, Denmark; http://www.cbs.dtu.dk/services/TMHMM/) (Möller et al., 2001; Nielsen et al., 2019). The Type N secretion system (TNSS) proteins were screened from the TNSS-associated proteins, which were chosen and filtered directly from multiple genomic functional databases, including Non-Redundant Protein database (NR; Li et al., 2002), Swiss-Prot database (Bairoch and Apweiler, 2000), Transporter Classification Database (TCDB; Saier et al., 2014), GO, PHI and VFDB. Type III secretion system (T3SS) effector was further predicted with EffectiveT3 software (version 1.0.1; University of Vienna, Vienna, Austria; https://effectors.csb.univie.ac.at/method/effectivet3), using the annotation results of the protein sequence function database according to the related proteins of the secretion system extracted and annotated (Jehl et al., 2011).

#### Genomic Visualization Analysis

The sequencing maps of the three selected strains were displayed using the Circos software (Canada’s Michael Smith Genome Sciences Centre, Vancouver, Canada; http://www.circos.ca/software/) after combining the prediction results of the encoded genes (Krzywinski et al., 2009).

### Survival Analysis

The three selected *V. parahaemolyticus* strains were cultured in 3% NaCl APW at 35°C, with shaking for 8 h, and the fresh culture suspensions were adjusted to OD values of 0.1, 0.2, and 0.3. The culture was centrifuged (1,680*g*, 10 min) and rinsed with sterile 9% NaCl three times, and the supernatant was discarded. The bacterial sediment was resuspended in 100 μl PBS to be made ready for intraperitoneal injection.

Eight-week-old female Balb/C mice with average weight of 18 g (17.4–18.6 g per each) were used for *in vivo* infection experiment. Survival assay contained nine groups, i.e., three selected strains with three different injection concentrations, and each test group was comprised of eight mice, allowed to feed and drink freely. The survival rate of each group was observed every hour for 12 h after *Vibrio* infection. After the 12-h acute infection period, the status of mice was observed every 12 h, and the number of deaths for each group was recorded. The experiment continued for a total of 4 days. After reaching the end of the *in vivo* experiment, the surviving mice were anesthetized excessively, and were cervical dislocated to death. The body of mice were put to harmless disposal after incineration.

All animal procedures complied with the institutional and national guidelines prescribed by the International Council for Laboratory Animal Science (ICLAS) from the Ministry of Health of the People’s Republic of China. All procedures performed in this work involving animals were in accordance with the ethical standards of Animal Ethics Committee of the Sixth Medical Centre of Chinese PLA General Hospital at which the work was conducted.

### Drug Sensitivity Test

A total of 22 different antimicrobial drug sensitivity test papers (OXOID, MA, USA) were used to perform the drug sensitivity tests for tetracycline (TE), cefotaxime (CTX), ceftazidime (CAZ), ciprofloxacin (CIP), levofloxacin (LEV), ofloxacin (OFX), ampicillin (AMP), amoxycillin/clavulanic acid (AMC), ampicillin/sulbactam (SAM), piperacillin (PRL), piperacillin/tazobactam (TZP), cephazolin (KZ), cefepime (FEP), cefoxitin (FOX), cefuroxime sodium (CXM), cephalothin (KF), imipenem (IPM), meropenem (MEM), amikacin (AK), gentamicin (CN), chloramphenicol (C), and sulphamethoxazole/trimethoprim (SXT). The Kirby-Bauer method, recommended by the Clinical and Laboratory Standards Institute, was used to determine the sensitivity of the 69 V*. parahaemolyticus* strains to the 22 antibiotics.

Single colonies of *V. parahaemolyticus* strains cultured on thiosulfate citrate bile salts sucrose agar culture medium (Beijing Land Bridge Technology, Beijing, China) for 18 h, were cultured in 3% NaCl APW at 35°C, with shaking for 8 h. The concentration of the culture was adjusted to 0.5 MCF with sterile physiological saline. A 90 mm Mueller-Hinton agar plate with 3% NaCl (Beijing Land Bridge Technology, Beijing, China) was streaked twice with swabs, and incubated for 20 min to affix the drug sensitivity test papers. Each plate was affixed with five different susceptibility test papers, and each antibiotic was tested in triplicate. After incubation at 35°C for 16 h, the bacteriostatic zone was measured with a Vernier caliper, and *E. coli* ATCC 25922 was used as a reference strain for quality control (**Table S1**).

### Statistics

Results were analyzed using one-way analysis of variance and Dunnett’s test by IBM Statistics SPSS (version 22) (IBM, NY, USA). *P* values were two-tailed, and the threshold for statistical significance was set at 0.05. Results are presented as mean ± standard error for all independent experiments at each time point. Kaplan-Meier method was used on survival analysis and Breslow test was used for statistical analyses by IBM Statistics SPSS (version 22) (IBM, NY, USA). Breslow *P* values for statistical significance was set at 0.05.

## Results

### General Attributes of All *V. parahaemolyticus* Strains and Screening of Representative Strains

Multiplex PCR results showed that all the strains of *V. parahaemolyticus* carried the *tlh* gene, encoding thermolabile hemolysin, specifically expressed by *V. parahaemolyticus*. Multiple combination types of Thermostable direct hemolysin- (TDH-) and thermostable related hemolysin- (TRH-) encoding genes were detected, as shown in **Table 1**. Among 69 strains, four (*Vp.* 1470, *Vp.* 1474, *Vp.* 1507, and *Vp.* 4215) carried the *tdh* gene and three (*Vp.* 1511, *Vp.* 1513 and *Vp.* 4213) carried the *trh* gene. However, none of the strains carried both *tdh* and *trh* at the same time.

**Table 1:** Hemolysin features of *V. parahaemolyticus* strains.

Strain ID	*tdh*	*trh*	*tlh*	Strain ID	*tdh*	*trh*	*tlh*	Strain ID	*tdh*	*trh*	*tlh*
*Vp.* 1463	−	−	+	*Vp.* 1464	−	−	+	*Vp.* 1465	−	−	+
*Vp.* 1466	−	−	+	*Vp.* 1467	−	−	+	*Vp.* 1468	−	−	+
*Vp.* 1469	−	−	+	*Vp.* 1470	+	−	+	*Vp.* 1471	−	−	+
*Vp.* 1472	−	−	+	*Vp.* 1473	−	−	+	*Vp.* 1474	+	−	+
*Vp.* 1475	−	−	+	*Vp.* 1476	−	−	+	*Vp.* 1477	−	−	+
*Vp.* 1478	−	−	+	*Vp.* 1479	−	−	+	*Vp.* 1480	−	−	+
*Vp.* 1481	−	−	+	*Vp.* 1482	−	−	+	*Vp.* 1483	−	−	+
*Vp.* 1484	−	−	+	*Vp.* 1485	−	−	+	*Vp.* 1486	−	−	+
*Vp.* 1487	−	−	+	*Vp.* 1488	−	−	+	*Vp.* 1489	−	−	+
*Vp.* 1490	−	−	+	*Vp.* 1491	−	−	+	*Vp.* 1492	−	−	+
*Vp.* 1493	−	−	+	*Vp.* 1494	−	−	+	*Vp.* 1495	−	−	+
*Vp.* 1496	−	−	+	*Vp.* 1497	−	−	+	*Vp.* 1498	−	−	+
*Vp.* 1499	−	−	+	*Vp.* 1500	−	−	+	*Vp.* 1501	−	−	+
*Vp.* 1502	−	−	+	*Vp.* 1503	−	−	+	*Vp.* 1504	−	−	+
*Vp.* 1505	−	−	+	*Vp.* 1506	−	−	+	*Vp.* 1507	−	−	+
*Vp.* 1508	−	−	+	*Vp.* 1509	−	−	+	*Vp.* 1510	−	−	+
*Vp.* 1511	−	+	+	*Vp.* 1512	−	−	+	*Vp.* 1513	−	+	+
*Vp.* 1514	−	−	+	*Vp.* 1515	−	−	+	*Vp.* 1516	−	−	+
*Vp.* 1517	−	−	+	*Vp.* 1518	−	−	+	*Vp.* 1519	−	−	+
*Vp.* 1520	−	−	+	*Vp.* 1521	−	−	+	*Vp.* 1522	−	−	+
*Vp.* 1523	−	−	+	*Vp.* 1524	−	−	+	*Vp.* 1525	−	−	+
*Vp.* 1526	−	−	+	*Vp.* 1527	−	−	+	*Vp.* 1528	−	−	+
*Vp.* 4213	−	+	+	*Vp.* 4215	+	−	+	*Vp.* 11577	−	+	+

**Figure 1:**
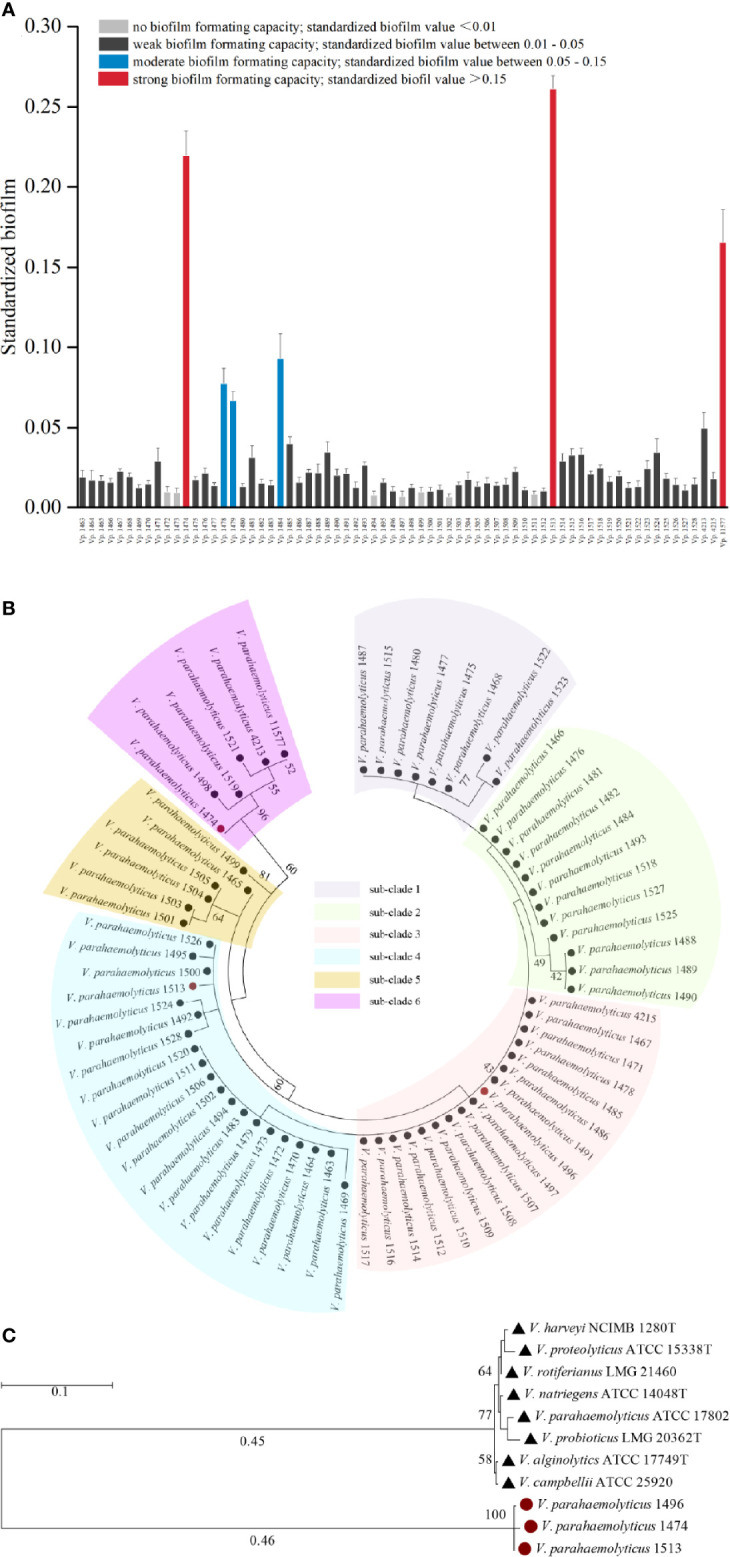
Phylogenetic analysis and biofilm formation capacity of *V. parahaemolyticus*. **(A)** Standardized biofilms formed by 69 V*. parahaemolyticus* environmental strains. Based on the standardized biofilm values, the biofilm formation (BF) capacity of the strains was divided into four groups, viz., strong, moderate, weak, and no capacity. **(B)** Maximum likelihood tree of 69 V*. parahaemolyticus* environmental strains based on the 16S rDNA phylogenetic analysis. The bootstrap percentage value was obtained from 1000 samplings. The 69 strains were divided into 6 subclades. **(C)** Maximum likelihood tree for *V. parahaemolyticus* strains *Vp*. 1474, *Vp*. 1496, and *Vp*. 1513 based on the 16S rDNA phylogenetic analysis using type strains in *Vibrio* genus as reference. The bootstrap percentage value was obtained from 1000 samplings. Red solid circle: selected strains of *Vp*. 1474, *Vp*. 1496, and *Vp*. 1513; black solid triangle: reference *Vibrio* strains.

Among the 69 strains of foodborne *V. parahaemolyticus*, only three (*Vp.* 1474, *Vp.* 1513, and *Vp.* 11577) could form strong biofilms and three (*Vp.* 1478, *Vp.* 1479, and *Vp.* 1484) could form medium level biofilms. Fifty-six of the remaining strains formed weak biofilms, and the remaining seven (*Vp.* 1472, *Vp.* 1473, *Vp.* 1494, *Vp.* 1497, *Vp.* 1499, *Vp.* 1502, and *Vp.* 1511) were unable to form a biofilm matrix. BF of the *V. parahaemolyticus* strains had been shown in **Figure 1A**.

The 16S rDNA phylogenetic tree showed that the 69 strains of foodborne *V. parahaemolyticus* belonged to six different phylogenetic branches (**Figure 1B**). It may be assumed that during evolution, a large evolutionary lineage difference occurred among these 69 isolates, resulting in a comparatively large difference in conserved gene sequences. Analyzing with the collaboration of the features of biofilm formation capacity and hemolysin expression, *Vp.* 1474, *Vp.* 1496, and *Vp.* 1513, each belonging to a different phylogenetic branch, were all showed significant phylogenetic differences to the reference strain *V. parahaemolyticus* ATCC 17802 (**Figure 1C**).

Therefore, *Vp.* 1474*^tdh^* ^+,^ *^trh^* ^−,BF+^, *Vp.* 1496*^tdh^* ^−,^ *^trh^* ^−,BF−^, and *Vp.* 1513*^tdh^* ^−,^ *^trh^* ^+,BF+^ were selected as the representative isolates to perform the experiments in this study.

### *De Novo* Sequencing Analysis of *V. parahaemolyticus*

#### Genome Assembly

Based on sequencing of small fragment library, basic genomic information such as genome size, heterozygosity and repeat rate could be obtained by 15-K-mer analysis as a rapid genome survey method. The basic evaluation of bacterial genome provided reference for the development of *de novo* sequencing strategy, and it contributed effective basis for subsequent protocol of genome assembly and annotation method of genome structure.

Based on 15-K-mer analysis, the original genome sizes of *Vp.* 1474, *Vp.* 1496, and *Vp.* 1513 were 5.34 Mb, 5.12 Mb and 5.62 Mb, respectively. After revision, the revised genome sizes of the three strains was 5.25 Mb, 5.03 Mb, and 5.53 Mb, respectively. The percentages of GC content in their genomes were 45.15%, 45.39%, and 45.28%, respectively. K-mer frequency distribution curve results of 15-K-mer analysis showed one main peak for all three strains, and the heterozygosity rates of all strains were less than 0.01%, as being 0%, 0.01% and 0.01% of *Vp.* 1474, *Vp.* 1496, and *Vp.* 1513, respectively (**Figure S1**).

#### Genome Component Analysis

**Figure 2:**
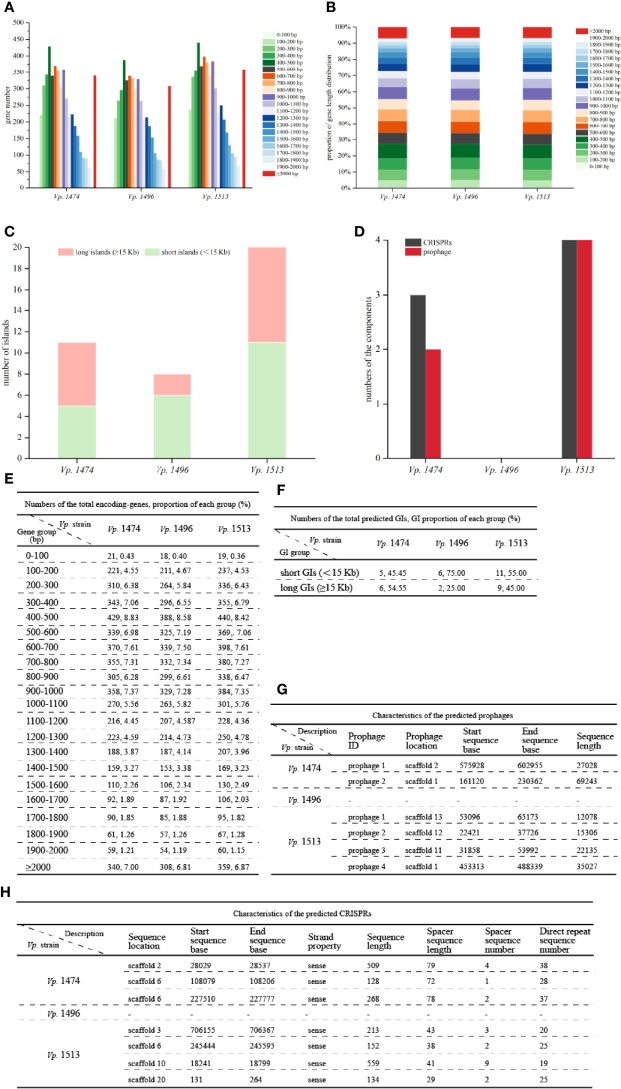
General genome components analysis of *V. parahaemolyticus* strains *Vp*. 1474, *Vp*. 1496, and *Vp*. 1513. **(A)** Statistical histogram of coding gene length distribution for the three strains. Coding gene zones were measured as 0 to 400 bp, 400 to 1,000 bp, 1,000 to 2,000 bp, and longer than 2000 bp as groups of short genes, medium long genes, long genes, and very long genes, respectively. **(B)** Percentage accumulative bar diagram of coding gene length distribution for the three strains. Coding gene zones were measured in 100 bp; 0 to 400 bp, 400 to 1,000 bp, 1,000 to 2,000 bp and longer than 2000 bp as groups of short genes, medium long genes, long genes and very long genes, respectively. **(C)** Accumulative bar diagram of genomic islands (GIs) for the three strains. GIs were divided into long islands and short islands by the length of 15 kb. **(D)** Statistical histogram of CRISPRs and prophages for the three selected strains. The component numbers of CRISPRs or prophages are shown on the Y-axis. **(E)** Statistical data of coding gene length distribution for the three strains. **(F)** Statistical data of GIs for the three strains. **(G)** Detailed characteristics of the predicted prophages for the three strains. **(H)** Detailed characteristics of the predicted CRISPRs for the three strains.

Analysis of the total encoding-gene levels from the genomes of *Vp.* 1474, *Vp.* 1496, and *Vp.* 1513 indicated that the total length of *Vp.* 1496 was the shortest, at 4249152 bp, whereas the total length of *Vp.* 1513 was the longest, at 4909851 bp; the total length of *Vp.* 1474 was 4539612 bp. However, the average lengths of gene sequences for *Vp.* 1474, *Vp.* 1496, and *Vp.* 1513 were nearly the same, at 934 bp, 940 bp, and 939 bp, respectively. From the distribution of gene lengths of the three strains, the gene length between 400 and 500 bp had the maximum proportion (8.83%, 8.58%, and 8.42% in the genome of *Vp.* 1474, *Vp.* 1496, and *Vp.* 1513, respectively), whereas the gene length between 0 and 100 bp had the minimum proportion (0.42%, 0.40% and 0.36% in the genome of *Vp.* 1474, *Vp.* 1496, and *Vp.* 1513, respectively; **Figures 2A, E**). The greatest gene length distribution differences between *Vp.* 1474, *Vp.* 1496, and *Vp.* 1513 were 800 to 900 bp (6.28%, 5.84% and 6.43% in the genome of *Vp.* 1474, *Vp.* 1496, and *Vp.* 1513, respectively), 400 to 500 bp (8.83%, 8.58%, and 8.42% in the genome of *Vp.* 1474, *Vp.* 1496, and *Vp.* 1513, respectively), and 500 to 600 bp (6.98%, 6.61% and 6.79% in the genome of *Vp.* 1474, *Vp.* 1496, and *Vp.* 1513, respectively) in descending order, which indicated a distribution diversity in the medium-long genes (**Figures 2B, E**).

GIs contained several genomic regions, which were integrated into bacterial genome by exogenous bacteria, phages or plasmids through HGT. Various of bacterial functions could be coded by GIs, which involved in bacterial symbiosis, pathogenic mechanisms, environmental adaptability, and so on. The IslandPath-DIOMB software was used as the predicting method. By detecting DNA and RNA phylogenetically bias and mobility genes existence, such as transposases and integrases, the potential HGT and GIs of *Vp.* 1474, *Vp.* 1496, and *Vp.* 1513 were predicted. Results for prediction of the total number of GIs for the three strains showed that the differences between these three strains were in the total numbers and the lengths of GIs (**Figures 2C, F**). *Vp.* 1513 was predicted to have the largest number of GIs (n = 20), followed by *Vp.* 1474 (n = 11), and *Vp.* 1496 (n = 8). Difference in the GIs of these strains was mainly reflected in the number and proportion of long GIs (≥ 15 Kb). There were separately six (GIs001, GIs002, GIs003, GIs006, GIs008, and GIs010) and nine (GIs004, GIs006, GIs008, GIs010, GIs011, GIs012, GIs016, GIs017, and GIs018) long GIs predicted in the genome of *Vp.* 1474 and *Vp.* 1513, which was separately 54.55% and 45% of the total predicted GIs, while only two long GIs (GIs005 and GIs006) were predicted in the genome of *Vp.* 1496, which was only 25% of the total predicted GIs. Detailed gene distribution in each GI for the three *Vp*. strains were shown in **Figure S2**.

CRISPR system was proved to be a bacterial adaptive immunity system, which showed crucial effect during invasion of viruses and plasmids. After the invading process, CRISPR locus could be integrated into host genome so that it caused the adaptive immunity for host to defense against subsequent attack by the same invader (Sun et al., 2015). Further study showed that CRISPR system partially reflected bacterial evolution. Based on the nomenclature initiated and optimized by Makarova et al., the CRISPR system existed in *V. parahaemolytius* belongs to the subtype I-F, encoded by cas gene *csy3* (Makarova et al., 2011). The number of CRISPRs and prophages in the genomes of *Vp.* 1474, *Vp.* 1496, and *Vp.* 1513 showed marked differences (**Figures 2D, G, H**). The genome of *Vp.* 1496 did not present any signs of CRISPR or prophage integration, and was thus considered as the most conservative. However, the genome of *Vp.* 1513 had four CRISPRs and four prophages, which was the highest number among these strains.

### Genome Function Analysis Based on Public Bioinformatics Databases

#### Genome Function Analysis Based on GO Database

**Figure 3:**
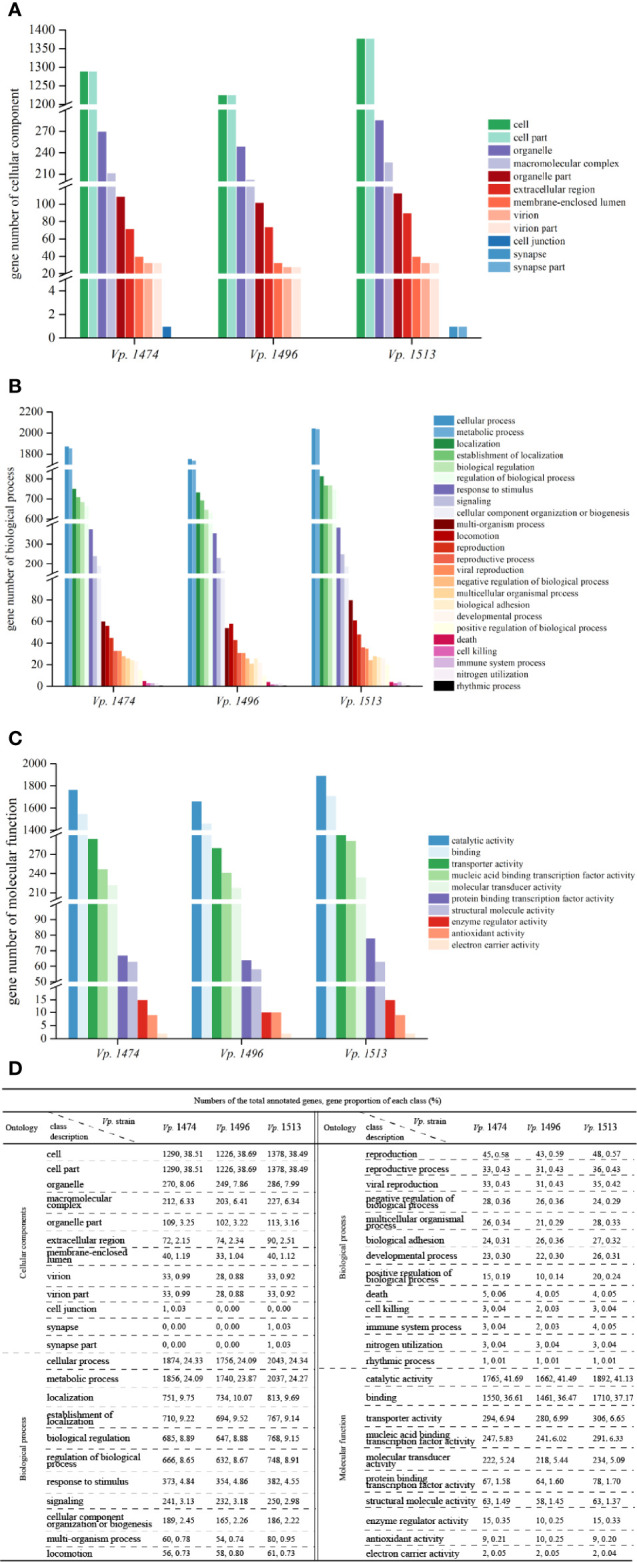
Gene ontology (GO) annotation of *V. parahaemolyticus* strains *Vp*. 1474, *Vp*. 1496, and *Vp*. 1513. **(A)** Statistical histogram of gene annotation to cellular component class of the three strains. A total of 13 parts were predicted in the genome of the three strains. Gene numbers are shown on the Y-axis. **(B)** Statistical histogram of gene annotation to biological process class of the three strains. A total of 24 parts were predicted within genome genes of the three strains. Gene numbers are shown on the Y-axis. **(C)** Statistical histogram of gene annotation to molecular function class of the three strains. A total of 10 parts were predicted within genome genes of the three strains. Gene numbers are shown on the Y-axis. **(D)** Statistical data of annotated genes based on GO ontology for the three strains.

By aligning with the GO database, more than 3000 genes were annotated in the genomes of *Vp.* 1474, *Vp.* 1496, and *Vp.* 1513. Moreover, there was a significant difference in the total annotation numbers and distributions among the three strains. For genes that annotated to the class of cellular components, *Vp.* 1513 had the greatest number of genes annotated, except for cell junction, whereas *Vp.* 1496 showed the opposite trend. *Vp.* 1474 had one gene annotated in cell junction component namely *Vp*. 1474 GM002208 that annotated with 24 GO accessions, whereas *Vp.* 1513 had one gene annotated in synapse or synapse part component namely *Vp*. 1513 GM002507 that annotated with 6 GO accessions. The components of extracellular region showed a different pattern between the three strains as compared with the trend that other components presented (**Figures 3A, D**).

For genes that annotated to the class of biological process, the total number of annotated genes was present in the same pattern as the class of cellular component. It was worth noticing that there were many more genes accounted for more proportion in the genome of *Vp.* 1513 involved in the multi-organism process (0.95% of total annotated genes) and multicellular organismal process (0.33% of total annotated genes), while *Vp.* 1496 showed the opposite trend (0.74% and 0.29% of total annotated genes, respectively). Meanwhile, the least number of genes (total number of 7288) in the genome of *Vp.* 1496 were involved in positive regulation of biological process (**Figures 3B, D**).

For genes that annotated to the class of molecular function, the total number of annotated genes in the genome of *Vp.* 1496 was the minimum among the three strains. The genes of *Vp.* 1513 were mostly found to be associated with catalytic activity, binding, and protein binding transcription factor activity, as in 41.13%, 37.17% and 1.70% of total annotated genes, respectively. On the contrary, the disadvantage of *Vp.* 1496 was mainly in the lack of genes that were involved in enzyme regulator activity (0.25% of total annotated genes; **Figures 3C, D**).

#### Genome Function Analysis Based on KEGG Database

**Figure 4:**
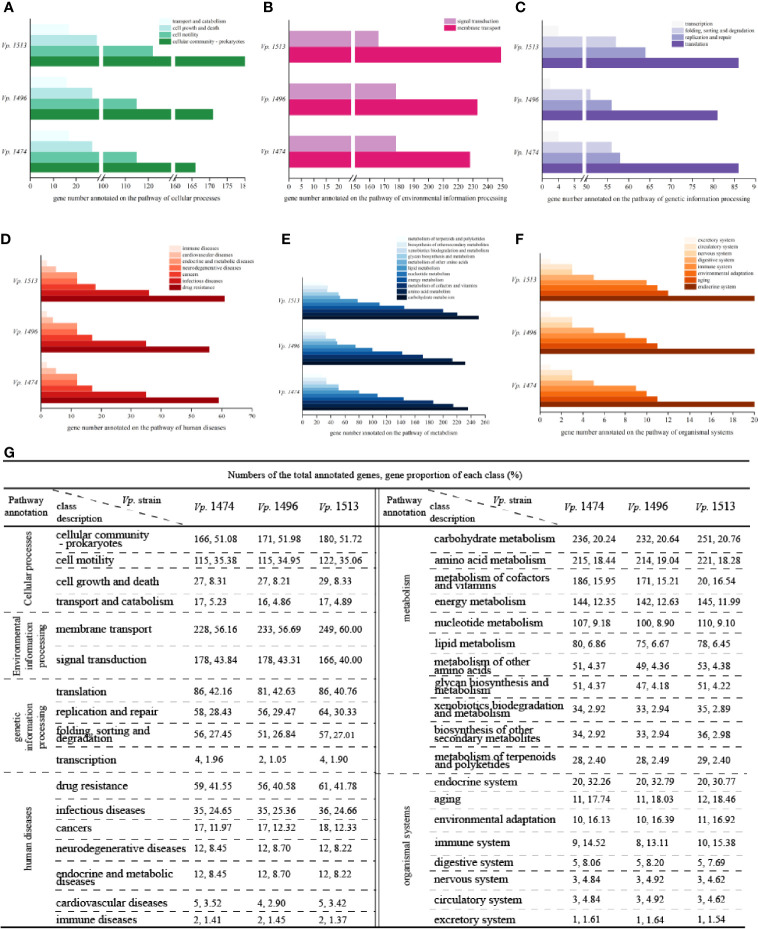
KEGG pathway annotation of *V. parahaemolyticus* strains *Vp*. 1474, *Vp*. 1496, and *Vp*. 1513. **(A)** Statistical bar diagram of genes predicted to cellular processes pathway of the three strains. A total of 4 pathways were predicted within genome genes of the three strains. Gene numbers are shown on the horizontal axis. **(B)** Statistical bar diagram of genes predicted for environmental information processing pathways in the three strains. A total of 2 pathways were predicted within genome genes of the three strains. Gene numbers are shown on the horizontal axis. **(C)** Statistical bar diagram of genes predicted for genetic information processing pathway of the three strains. A total of 4 pathways were predicted within genome genes of the three strains. Gene numbers are shown on the horizontal axis. **(D)** Statistical bar diagram of genes predicted to human diseases pathways of the three strains. A total of 7 pathways were predicted within genome genes of the three strains. Gene numbers are shown on the horizontal axis. **(E)** Statistical bar diagram of genes predicted to metabolism pathways of the three strains. A total of 11 pathways were predicted within genome genes of the three strains. Gene numbers are shown on the horizontal axis. **(F)** Statistical bar diagram of genes predicted to organismal systems pathways of the three strains. A total of 8 pathways were predicted within genome genes of the three strains. Gene numbers are shown on the horizontal axis. **(G)** Statistical data of KEGG pathway annotation for the three strains.

By Aligning with the KEGG database, 4644, 4391, and 5017 genes were annotated in the genomes of *Vp.* 1474, *Vp.* 1496, and *Vp.* 1513, respectively. For encoding genes that participate in the pathway of cellular processes, the numbers of genes for *Vp.* 1513 annotated to cellular community, cell motility, and cell growth and death were significantly higher than that of *Vp.* 1474 and *Vp.* 1496. Considering the genome size, the proportion of the gene numbers of *Vp.* 1496 annotated to cellular community (51.98% of total annotated genes) was considerably more than that of *Vp.* 1474 (51.08% of total annotated genes) and *Vp.* 1513 (51.72% of total annotated genes). Despite of the higher gene proportion of *Vp.* 1496 annotated to transport and catabolism (5.23% of total annotated genes) than it of *Vp.* 1474 (4.86% of total annotated genes) and *Vp.* 1513 (4.89% of total annotated genes), the gene numbers for these three strains tended to be equal (**Figures 4A, G**).

For genes that are associated with the pathway of environmental information processing, there was a difference in distribution between the class of membrane transport and signal transduction. Although the genome size of *Vp.* 1496 was the least among these strains, it had more genes annotated to the membrane transport class (56.69% of total annotated genes) than *Vp.* 1474 (56.16% of total annotated genes) and more annotated to the signal transduction class (43.31% of total annotated genes) than *Vp.* 1513 (40% of total annotated genes; **Figures 4B, G**). For genes that were homologous to the cluster of genetic information processing, the gene numbers of *Vp.* 1513 were annotated the most. Note that the gene numbers of *Vp.* 1474 and *Vp.* 1513 annotated to the pathways of translation, folding, sorting, degradation, and transcription were almost equal. However, there were more genes in the *Vp.* 1513 genome participating in the pathway of replication and repair (**Figures 4C, G**). For genes that were participating in the pathway of human diseases, the main difference between the annotated genes for *Vp.* 1474, *Vp.* 1496, and *Vp.* 1513 was the number for drug resistance class, which showed a significantly lower number and a lower proportion of genes in the genome of *Vp.* 1496 (40.58% of total annotated genes**;** **Figures 4D, G**).

There was an abundance of genes participating in the pathway of metabolism; this number was the largest among the five clusters from the KEGG database. Among the 11 classes in this cluster, carbohydrate metabolism was the class that had the highest number and the highest proportion (20.24%, 20.64% and 20.76% of total annotated genes in *Vp.* 1474, *Vp.* 1496 and *Vp.* 1513, respectively) of annotated genes. Among the three strains, *Vp.* 1513 had the most genes annotated, and *Vp.* 1496 had the least. However, in the class of metabolism of terpenoids and polyketides, the numbers of annotated genes were almost the same between these strains (**Figures 4E, G**). Similarly, there was hardly any difference in the numbers of annotated genes participating in the pathway of organismal systems (**Figures 4F, G**).

#### Genome Function Analysis Based on COG Database

**Figure 5:**
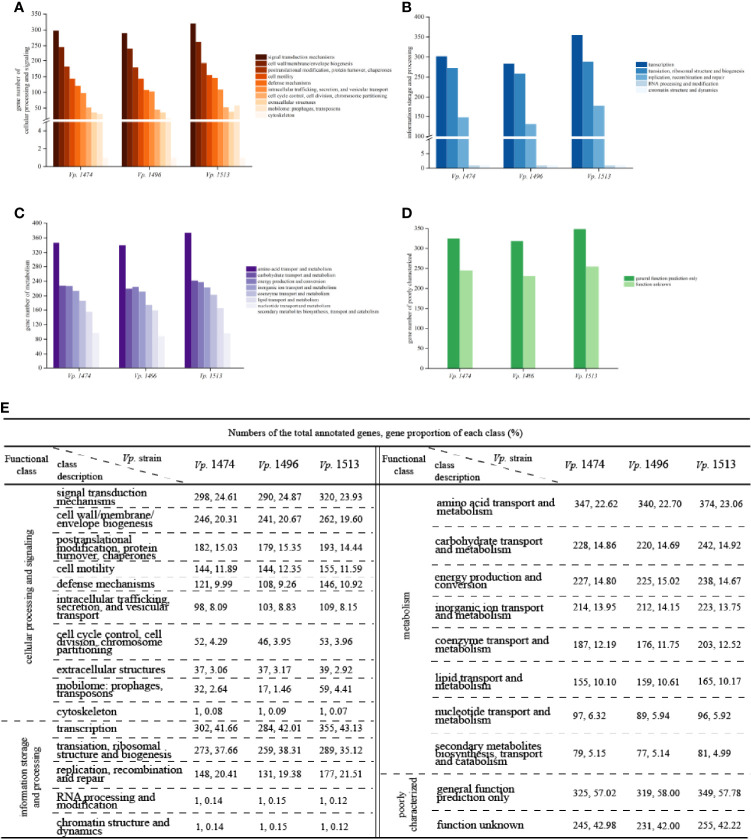
COG function classification of *V. parahaemolyticus* strains *Vp*. 1474, *Vp*. 1496, and *Vp*. 1513. **(A)** Statistical histogram of gene classified to cellular processing and signaling cluster of the three strains. A total of 10 sub-clusters were predicted within genome genes of the three strains. Gene numbers are shown on the Y-axis. **(B)** Statistical histogram of gene classified to information storage and processing cluster of the three strains. A total of 5 sub-clusters were predicted within genome genes of the three strains. Gene numbers are shown on the Y-axis. **(C)** Statistical histogram of gene classified to metabolism cluster of the three strains. A total of 8 sub-clusters were predicted within genome genes of the three strains. Gene numbers are shown on the Y-axis. **(D)** Statistical histogram of poorly classified genes of the three strains. Only one sub-cluster was predicted to have general function, and the functions of the remaining genes were unknown. Detailed gene numbers are shown on the Y-axis. **(E)** Statistical data of annotation based on COG database for the three strains.

Alignment with the COG database indicated that the numbers of annotated genes were similar to those for the GO database, at 3553, 3411, and 3857 for the genomes of *Vp.* 1474, *Vp.* 1496, and *Vp.* 1513, respectively, which meant that these three genomes have significant differences in protein-coding genes. For genes that were homologous to the cluster of cellular processing and signaling, the significant differences of protein-encoding genes were in the class of defense mechanisms and mobilome, with *Vp.* 1513 having the greatest number and proportion (10.92% and 4.41% of total annotated genes, respectively) and *Vp.* 1496 having the least (9.26% and 1.46% of total annotated genes, respectively; **Figures 5A, E**).

For genes that were homologous to the cluster of information storage and processing, the higher phylogenetic evolution level of *Vp.* 1513 was reflected in its high number and proportion of genes for replication, recombination, and repair (21.51% of total annotated genes), as well as dynamic transcription activity (43.13% of total annotated genes; **Figures 5B, E**). For genes that were homologous to the cluster of metabolism, there was no significant difference between the three strains (**Figures 5C, E**). Furthermore, there were over 550 genes with unknown functions on the basis of COG annotation results (**Figures 5D, E**).

### Comparative Analysis of *V. parahaemolyticus* Virulence and Pathogenicity

**Figure 6:**
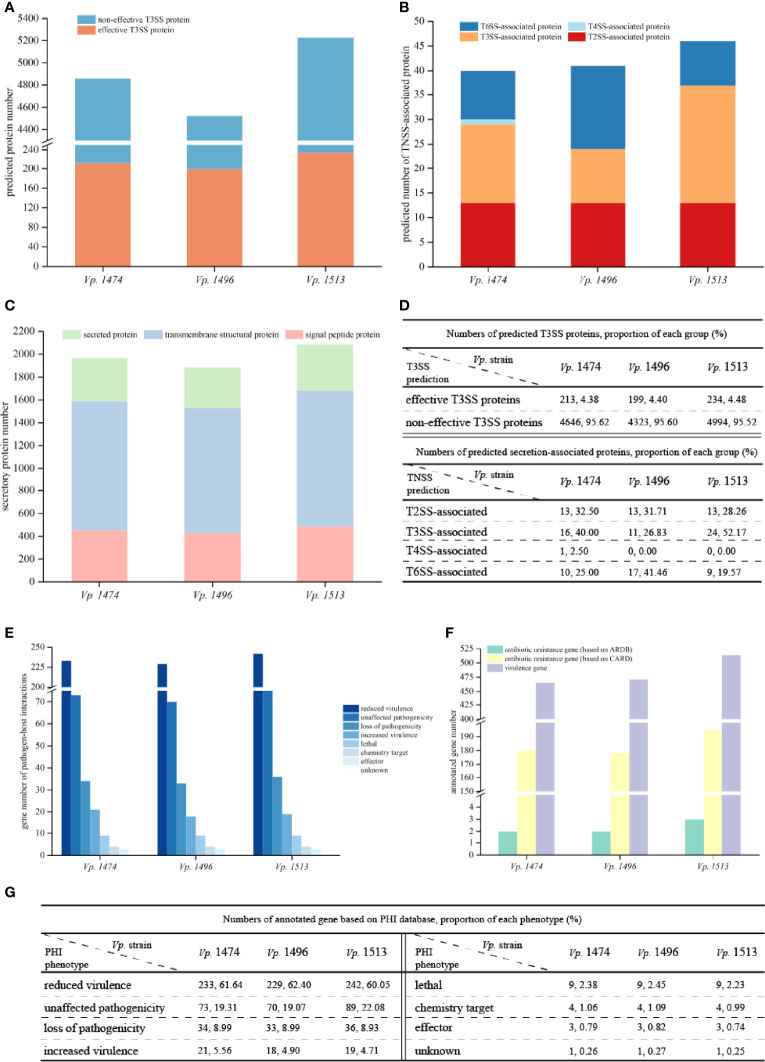
Virulence and pathogenicity analysis of *V. parahaemolyticus* strains *Vp*. 1474, *Vp*. 1496, and *Vp*. 1513. **(A)** Accumulative bar diagram of protein distribution in Type III secretion system (T3SS) of the three strains. T3SS were predicted and divided into effective and non-effective proteins, and their numbers are shown on the Y-axis. **(B)** Accumulative bar diagram of protein distribution in major Type N secretion system (TNSS) of the three strains. Four major TNSS were predicted in the three strains, viz., T2SS, T3SS, T4SS, and T6SS, and the number of TNSS-associated proteins are shown on the Y-axis. **(C)** Accumulative bar diagram of secretory proteins distribution of the three strains. Secretory proteins were divided into 3 groups, namely secreted proteins, transmembrane structural proteins, and signal peptide proteins. Numbers and detailed distribution of proteins are shown on the Y-axis. **(D)** Statistical data of predicted T3SS and other secretion-associated proteins for the three strains. **(E)** Statistical histogram of genes that are involved in pathogen-host interaction of the three strains. A total of 8 classes were predicted within genome genes of the three strains. Gene numbers are shown on the Y-axis. **(F)** Statistical histogram of genes annotated to antibiotic resistance and virulence of the three strains. Genes associated with antibiotic resistance were predicted and aligned with CARD and ARDB databases; genes annotated to virulence were predicted and aligned with VFDB database. Detailed numbers are shown on the Y-axis. **(G)** Statistical data of gene annotation based on PHI database for the three strains.

T3SS prediction was performed using EffectiveT3 software, and the predicted sequences were scored and screened for the final determination of T3SS effective proteins. Although the predicted number of T3SS effective proteins for *Vp.* 1496 was the least (n=199) whereas the number of that for *Vp.* 1474 and *Vp.* 1513 were 213 and 234, respectively, their proportion compared to other proteins was not the lowest among the three selected strains, as in 4.38%, 4.40% and 4.48% of total annotated proteins, respectively (**Figures 6A, D**).

Based on the results of gene sequences, there were three common secretion systems in the genomes of *Vp.* 1474, *Vp.* 1496, and *Vp.* 1513, including Type II secretion system (T2SS), T3SS, and Type VI secretion system (T6SS), whereas only *Vp.* 1474 had the Type IV secretion system (T4SS). Three strains all presented 13 T2SS-associated proteins. However, *Vp.* 1496 presented 17 T6SS-associated proteins (41.46% of total annotated proteins), which was significantly more than the corresponding values of 10 and 9 for *Vp.* 1474 (25% of total annotated proteins) and *Vp.* 1513 (19.57% of total annotated proteins), respectively (**Figures 6B, D**). In addition to TNSS-associated proteins, secreted proteins are another important type of protein that enhance *V. parahaemolyticus* pathogenicity. It was observed that for *Vp.* 1513, the numbers of signal peptide proteins and secreted proteins were similar and less than the number of transmembrane structural proteins. However, for transmembrane structural proteins, *Vp.* 1513 was predicted to have the lowest proportion of total annotated proteins (57.33%), whereas *Vp.* 1496 was predicted to have the highest proportion (58.55%; **Figures 6C, D**). Based on the interpretation of NR and GO databases, the difference of T3SS-associated proteins among the three strains reflected on the difference of number and types of both apparatus proteins and accessory proteins such as secretion protein, membrane protein, contacting sensing protein and so on. *Vp*. 1474 had the greatest number of annotated apparatus proteins (n=4), *Vp*. 1496 had the least number (n=1), and *Vp*. 1513 had 2 apparatus proteins. Combining with the GO interpretation, *Vp*. 1496 lacked 3 apparatus proteins that might be involved in the function of ATPase activity, actin binding and proton transporting.

Based on the PHI database, the number of genes in the genomes of *Vp.* 1474, *Vp.* 1496, and *Vp.* 1513 annotated on lethal, chemistry target, and effector were the same. Associated with pathogen-host interaction, one gene existed in all three strains (*Vp*. 1474 GM000792, *Vp*. 1496 GM000568, and *Vp*. 1513 GM000720, respectively) with unclassified function, which might be identified as the similar function of gene *feoB* (PHI-base accession number 6941) that involved in the coding of ferrous iron transport protein (UniProtKB accession: A0A0F6UGK4). *Vp.* 1513 had the greatest number of genes annotated by this method, whereas *Vp.* 1496 had the least number of annotated genes. However, *Vp.* 1474 had the greatest number and the highest proportion (5.56%) of genes that were annotated in the class of increased virulence, and *Vp.* 1496 had the highest proportion of genes (2.45%) annotated in the class of lethal (**Figures 6E, G**). Based on the VFDB, *Vp.* 1513 had more genes annotated than *Vp.* 1474 and *Vp.* 1496 (**Figure 6F**), indicating a significantly higher virulence potential of this strain.

Based on the ARDB, the three strains had a slight difference in antibiotic resistance, with two to three genes responding to drug resistance. However, there were more than 170 drug resistance genes in the genomes of *Vp.* 1474, *Vp.* 1496, and *Vp.* 1513 based on the CARD. *Vp.* 1513 had the largest gene number annotated for drug resistance, whereas *Vp.* 1474 and *Vp.* 1496 had fewer, but similar number of genes annotated in that database (**Figure 6F**).

### Genome-Wide Map of *V. parahaemolyticus*

**Figure 7:**
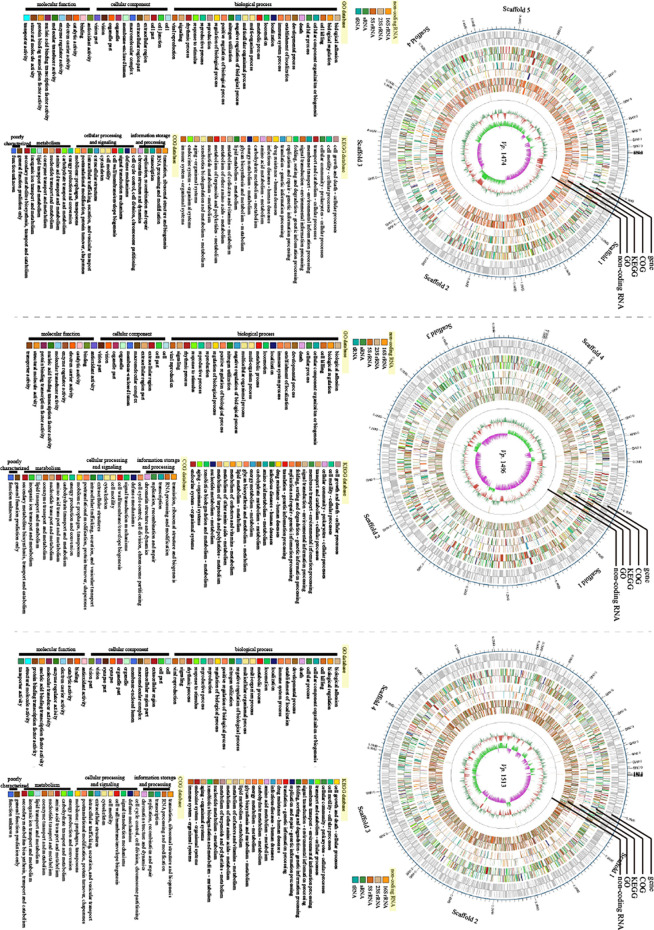
Genomic visualization analysis of *V. parahaemolyticus* strains *Vp*. 1474, *Vp*. 1496, and *Vp*. 1513. Three genome-wide maps represented the combination of the prediction results of the coding genes. Results of gene distribution analysis, COG classification, KEGG pathway, GO annotation, and non-coding RNA are presented from the outermost to innermost rings.

Based on the assembled genome sequences of *Vp.* 1474, *Vp.* 1496, and *Vp.* 1513, the circular structures of the genomes of these three strains were displayed in a genome-wide map, with the combination of the respective prediction results of the coding genes. In the genome-wide map, relational analysis results were also displayed, including those obtained using non-coding RNA, KEGG, GO, and COG databases, which predicted the gene function annotations of *V. parahaemolyticus* (**Figure 7**).

### *V. parahaemolyticus* Pathogenicity *In Vivo*

**Figure 8:**
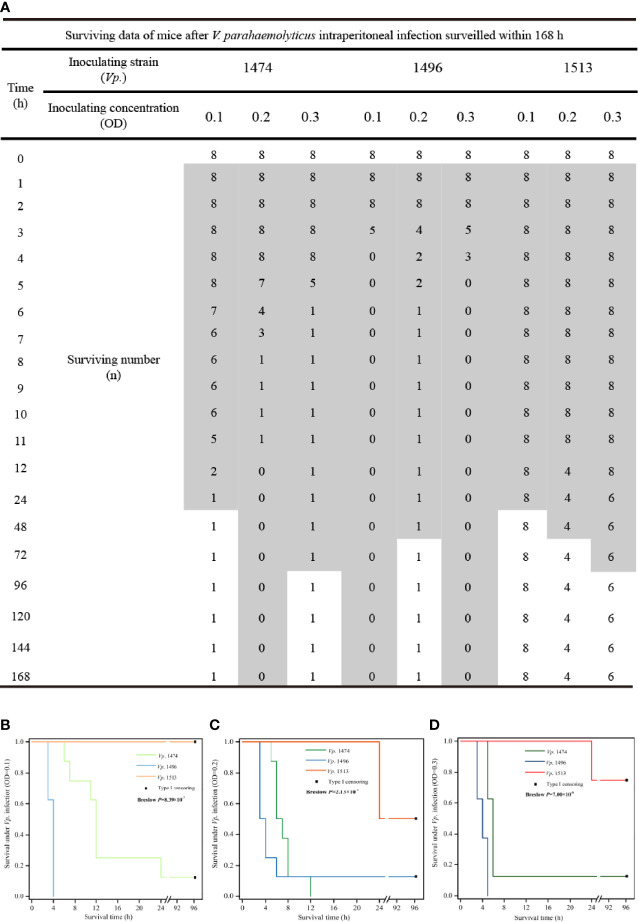
*V. parahaemolyticus in vivo* infection and data statistics. **(A)** Operation of *in vivo* infection and data presentation. Suspension cultures of the selected strains, viz., *Vp*. 1474, *Vp*. 1496, and *Vp*. 1513, were adjusted to optical densities (OD) of 0.1, 0.2, and 0.3, and intraperitoneally injected into each group (9 groups, 8 mice per group). **(B)** Survival analysis of mice infected with *V. parahaemolyticus* under low concentration of injection (OD=0.1). **(C)** Survival analysis of mice infected with *V. parahaemolyticus* under moderate concentration of injection (OD=0.2). **(D)** Survival analysis of mice infected with *V. parahaemolyticus* under high concentration of injection (OD=0.3). Grey patch: the surviving mice that were observed in a poor living condition. The survival rate was updated every hour in acute infection period (≤12 h), and subsequently updated every 12 h in infection recovery period. The experiment continued for 4 days.

The theoretically predicted difference in pathogenicity for the selected strains, *Vp.* 1474, *Vp.* 1496, and *Vp.* 1513, was confirmed by the intraperitoneal infection model in mice, and the survival condition was showed as data and survival analysis in **Figure 8**. After 1 h of inoculation, the mice all showed different degrees of lethargy, inappetence, accomplishing with rough and messy hair. According to our observations, the course of disease was short after *Vp.* 1474 infection, and subjects could recover within 36 h. However, after *Vp.* 1496 infection, the course of disease was longer than that after *Vp.* 1474 infection, and subjects could return to health after 48 h. Conversely, after *Vp.* 1513 infection, the course of disease was the longest, and took a minimum of 96 h for the subjects to restore their health (**Figure 8A**).

Comparing the three chosen *V. parahaemolyticus*, the most serious acute infection induced by *Vp.* 1496 were observed within 2 h even under a low bacterial inoculate concentration (OD=0.1; **Figure 8B**), whereas the acute infection caused by *Vp.* 1474 and *Vp.* 1513 was showed after 5 h or 6 h positively related to bacterial inoculate concentration (**Figures 8C, D**). *Vp.* 1496 had the highest fatality rate after infection, the fatality of *Vp.* 1474 was closely related to the concentration of bacteria administered, and *Vp.* 1513 had the lowest fatality.

After at least 2 d and up to 4 d of inoculation, the surviving mice gradually returned to normal posture; however, the final survival after intraperitoneal infecting by different *V. parahaemolyticus* was significantly different (*P*<0.001) based on statistical analyses despite of the inoculating concentration (**Figures 8B–D**), and final survivors after *Vp.* 1513 infection retained the most. The results of survival analysis of the mice also showed that the different *V. parahaemolyticus* strains had different pathogenic effects on infected mice, with significant differences in infection rate and mortality.

### Antibiotic Resistance of *V. parahaemolyticus* Strains

**Figure 9:**
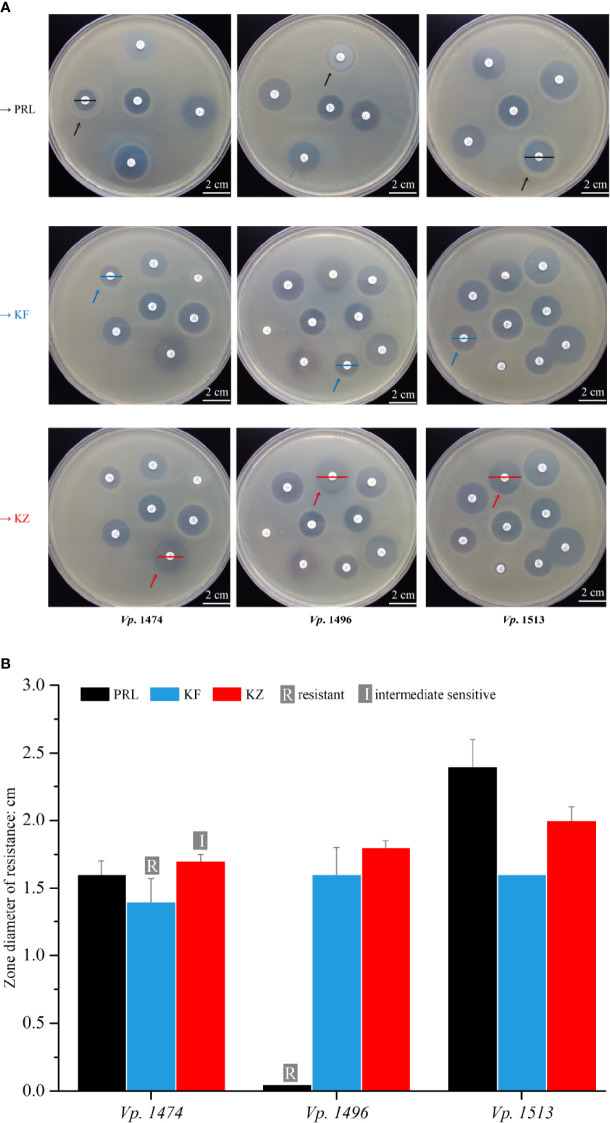
Antibiotic resistance analysis of mice infected by *V. parahaemolyticus* strains *Vp.* 1474, *Vp.* 1496, and *Vp.* 1513. **(A)** Representative results of antimicrobial susceptibility test using Kirby-Bauer method. Black arrow: paper position of piperacillin (PRL) on the representative plates; blue arrow: paper position of cephalothin (KF) on the representative plates; red arrow: paper position of cephazolin (KZ) on the representative plates; black line: zone diameter of *V. parahaemolyticus* strains resistant to PRL; blue line: zone diameter of *V. parahaemolyticus* strains resistant to KF; red line: zone diameter of *V. parahaemolyticus* strains resistant to KZ. **(B)** Statistical histogram of antibiotic resistance to PRL, KF, and KZ for the three strains. For PRL, the zone diameters for resistant, intermediately sensitive, and sensitive are ≤1.7 cm, 1.8 to 2.0 cm, and ≥2.1 cm, respectively; for KF and KZ, the zone diameters for resistant, intermediately sensitive, and sensitive are ≤1.4 cm, 1.5 to 1.7 cm, and ≥1.8 cm, respectively.

In order to explore the concordance between biofilm formation capacity and the antibiotic resistance, 22 antibiotics that are frequently used clinically were tested against the 69 V*. parahaemolyticus* strains. Among the antibiotics for which resistance was tested, the 69 strains of *V. parahaemolyticus* were all sensitive to 16 antibiotics, but the zone diameter values (cm) were ambiguous for two out of 16 (data not shown). Focusing on the three selected strains, *Vp.* 1496 was significantly resistant to PRL (mean zone diameter value=0.05 cm), *Vp.* 1474 was resistant to KF (mean zone diameter value=1.4 cm) and was intermediately sensitive to KZ (mean zone diameter value=1.7 cm), and *Vp.* 1513 was sensitive to PRL, KF, and KZ (**Figure 9A**). According to the zone diameter values, although the definition of drug resistance feature was different between KF and KZ resistance in *Vp*. 1474, the zone diameters were only marginally different (**Figure 9B**). It was worth noting that all 69 strains were resistant to AMP. Results indicated that the nature of drug resistance did not differ significantly amongst the different environmental strains of *V. parahaemolyticus*, and certain strains had gradually developed multi-antibiotic resistance potential.

## Discussion

Due to the increasing global incidence of foodborne gastroenteritis caused by *V. parahaemolyticus* (Rezny and Evans, 2020), it is imperative to reevaluate the pathogenicity of this bacteria using modern tools. WGS has played an important role in analyzing the evolution of *V. parahaemolyticus* and revealing the mechanism of their rapid serotype conversion (Han et al., 2019). However, the uncertainty of pathogenic difference on *V. parahaemolyticus* remains the difficulty providing complication to its virulence and pathogenic prediction, which reflects in the different isolating sources, geographic positions and infecting concentration correlation. Therefore, this study focused on the interpretation on the genome differences of three environmental *V. parahaemolyticus* namely *Vp*. 1474, *Vp*. 1496, and *Vp*. 1513. Results obtained by this study could provide the possible explanation to the pathogenic and virulent difference in environmental isolates.

### Genome Component Analysis for CRISPR, Prophage, and GIs

According to the *de novo* sequencing results in our study, there was a significant difference in the number of genes with different lengths in the genomes of *Vp.* 1474, *Vp.* 1496, and *Vp.* 1513, which indicated the existence of distinct evolutionary trajectories for the three *V. parahaemolyticus* strains. Compared to *Vp.* 1496, there was a higher number of long genes in the genome of *Vp.* 1513, indicating its significant evolutionary advantages.

#### CRISPR

CRISPR is a part of the prokaryotic adaptive immune system, which evolves when bacteria defend themselves against exogenous DNA, such as during phage attacks (Labrie et al., 2010). It consists of direct repeat, leader sequence, spacer sequence, and CRISPR associated (Cas) protein (Wright et al., 2016). The rapid evolution of bacteria depends on the insertion of exogenous spacer sequences into the genome to increase the chances of survival (Barrangou et al., 2007). Analyzing the genes annotated to CRISPR elements of the selected strains revealed that *Vp.* 1513 had the most CRISPR elements, whereas *Vp.* 1496 did not show any genes annotated to CRISPR elements integrated in its genome. The difference of CRISPR number in the genomes of *V. parahaemolyticus* environmental isolates might relate to genus specificity and *V. parahaemolyticus* source (Skovgaard et al., 2001). Based on the phylogenetic results showed in **Figure 1B**, *Vp.* 1513 underwent a higher level of genetic variation than that of *Vp.* 1496, and proceeded a longer evolutionary process, therefore the integration of a higher number of CRISPR elements might be due to phage attacks improved its resistance against viruses. In addition, studies confirmed that CRISPR was involved in biological behaviors of bacteria such as biofilm formation and swarming motility, besides self-defense (Zegans et al., 2009). The presence of a higher number of CRISPR elements might explain why *Vp.* 1513 had stronger biofilm formation capacity than other tested *V. parahaemolyticus* strains.

#### Prophage

Prophages integrate into the genome of bacterial hosts and pass along with bacterial proliferation, the feature which determines HGT. Thus, prophages often have an important effect on the pathogenicity of bacteria (Zrelovs et al., 2020). Takashi et al. reported a negative correlation between the number of prophages and CRISPRs in *Streptococcus pyogenes*, indicating that CRISPR integration could limit the insertion of prophage (Nozawa et al., 2011). Comparative genomic research showed that pathogenic *V. parahaemolyticus* could promote the insertion of prophage when lacking CRISPR (Yu et al., 2020).

Results of analysis for prophage genes in the genomes of *Vp.* 1474, *Vp.* 1496, and *Vp.* 1513 indicated that *Vp.* 1513 had the highest number of prophage genes, while *Vp.* 1496 had none. This phenomenon was inconsistent with the reports in literature. The difference between our research and previous literature might be explained by the concept that multiple survival advantages provided by lysogenic infection by prophage in *V. parahaemolyticus* could enable the survival of bacteria in an adverse environment (Wang et al., 2010).

#### GIs

GIs expand gene diversity through autologous transfer and loss of genetic information and participate in the genetic evolution of a microorganism. Notably, GIs arising due to HGT were a major reason for the evolution of novel pathogenic *Vibrio* strains (Hazen et al., 2010; Deng et al., 2019). GIs in the genome of *V. parahaemolyticus* are referred to as *Vibrio* pathogenicity islands for encoding virus factors and are a major reason for the evolution of novel pathogenic *V. parahaemolyticus* strains (Hacker and Carniel, 2001).

In this study, two out of three environmental isolates of *V. parahaemolyticus* had GIs in their genomes, confirming the phenomenon of HGT present in the selected *V. parahaemolyticus*. It could actuate *V. parahaemolyticus* environmental isolates to evolve towards pandemic strains by enhancing the differences of GIs and other mobile elements in the genome (Chen et al., 2011), and subsequently enhance the virulence potential by increasing the adaption of environmental isolates to marine environment (Baker-Austin et al., 2017), and finally acquiring the capacity to infect humans. The above-mentioned evidence indicated that *V. parahaemolyticus* might had upgraded their pathogenicity potential. However, it is difficult to explain this phenomenon only based on SNP and HGT analysis. Therefore, it needs to be compared on the whole genome level while retrospecting the evolution of *V. parahaemolyticus* to assess the extent and type of changes (Loyola et al., 2016).

### Genome Function Analysis Based on Public Bioinformatics Databases

In our study, research focusing on the genomes of *Vp.* 1474, *Vp.* 1496, and *Vp.* 1513 was performed through annotation using a combination of databases. According to our results, there were distinct differences in the functional annotations of biological process (GO database), organismal systems (KEGG database), and information storage and processing (COG database).

Based on the interpretation of GO database, *Vp*. 1496 lacked genes that was involved in the construction of membrane and synaptic transmission, as the performance of gene GM002208 in *Vp*. 1474 genome or gene GM002507 in *Vp*. 1513 genome. Existing in a type of septin-associated protein, *Vp*. 1474 GM002208 formed as a ring-shaped structure and involved in the formation of BLOC-1 complex, so that bacterial cells could adhere and then provide a discrete opening to eukaryotic cell of hosts by joining the nuclear membranes as a transport vesicle. *Vp*. 1474 GM002208 could be built and secreted as a transport vesicle in Golgi to perform the function of tyrosine kinase and transporter activity, involving in lipid and DNA binding, transcription elongation regulation, protein phosphorylation and metabolic processes. *Vp*. 1513 GM002507 encoded a part of postsynaptic membrane. Through the binding of zinc ion and acetylcholine receptor, *Vp*. 1513 GM002507 involved in synaptic transmission.

Annotating to subclass of multi-organism and multicellular organismal processes, *Vp*. 1496 showed a serious lack of genetic constitution compared to *Vp*. 1513 both in gene number and gene proportion, which presented a less of its genome in these functions. In addition, the genome of *Vp.* 1496 lacked genes that were related to interaction between bacteria and host, which was reflected by the significant decrease of enzyme numbers that regulated this function. The reason for this may be the reduced protein transcriptional activity, which was in accordance with the lack of genes that involved in transportation and synaptic transmission by the explanation of GO database.

Gene numbers annotated to the drug resistance subclass for the class of human disease in the KEGG database were distinct, possibly accounting for the large differences in antibiotic resistance among the three strains, especially for piperacillin. According to the gene distribution for the class of genetic information processing and cellular processes in KEGG database, gene numbers of *Vp.* 1513 that annotated to subclass of replication and repair, cellular community—prokaryotes, and cell motility were significantly more than that of *Vp.* 1474 and *Vp.* 1496. This indicated that the mechanisms of cell proliferation and repairing in *Vp.* 1513 were more complicated, resulting in enhanced bacterial communication and cell motility, which could help *Vp.* 1513 to escape from surrounding adverse environment and seek more advantageous environments for colonization, ultimately increasing its chances of survival. On the contrary, the evolutionary process of *Vp.* 1496 was more conservative, and there was a lack of interconnection and coordinated communication between bacterial populations, resulting in the lack of adaptability to harsh environments.

Nevertheless, the virulence of *Vp*. 1496 was significantly increased based on the *in vivo* infection results. According to the results interpretation on GO and KEGG databases, a significant larger number and proportion of genes of the genome of *Vp*. 1496 was annotated and classified in subclasses of locomotion (58, 0.80% in the genome) and biological adhesion (26, 0.36% in the genome) than those in genome of *Vp*. 1474 and *Vp*. 1513, in addition with more proteins involved in the signal pathways of prokaryotic cellular community and digestive system. These results revealed that the increased virulence of *Vp*. 1496 possibly depended on the precursor process of quorum communication-derived cell adhesion, which regulated the movement of bacterial cells and their adherence towards suitable hosts and suitable proliferating environment such as digestive system.

### *V. parahaemolyticus* Pathogenicity (TDH or TRH/TNSS)

Previous studies confirmed that various biological processes play significant roles in the pathogenicity of *V. parahaemolyticus*, such as hemolysis. TDH and TRH are two major hemolysins involved in *V. parahaemolyticus* pathogenicity, due to their distinct cell cytotoxicity and hemocytocatheresis (Cai and Zhang, 2018). The analysis of the expression of hemolysin genes is an important approach for understanding the pathogenicity of *V. parahaemolyticus*.

Considering that the genome of foodborne *V. parahaemolyticus* strains contained various functional regions with rich diversity, the differences in pathogenicity may have resulted from the distinction in the distribution of key genetic elements and differences in the secretory system compositions.

#### TDH or TRH

Compared with environmental isolates, clinical isolates of *V. parahaemolyticus* usually carry virulence factors, such as TDH or TRH (Nair et al., 2007), which may increase the toxicity of *V. parahaemolyticus* carrying both TDH and TRH (Martinez-Urtaza et al., 2017). Although, most *V. parahaemolyticus* are non-pathogenic isolates (Nair et al., 2007), even non-toxic *V. parahaemolyticus* can cause acute gastroenteritis (Ottaviani et al., 2012). Clinical isolates lacking TDH and TRH, such as the *V. parahaemolyticus* ST674 strain (Xu et al., 2017), have also been reported to have cytotoxicity independent of the TDH or TRH production (Lynch et al., 2005), indicating that non-toxic *V. parahaemolyticus* might also possess novel virulence mechanisms (Kamruzzaman et al., 2008). Previous studies could explain the results of mice infection model in our study. In our research, although the expression of TDH or TRH was detected in various strains, none of the 69 strains expressed TDH and TRH at the same time, indicating that *V. parahaemolyticus* environmental isolates expressed hemolysin at a low level, with a much lower level of expression of multiple hemolysins in the population. This conclusion was coincident with the previous study (Jiang et al., 2019), which might be a key trend of the distribution of hemolysin from *V. parahaemolyticus* environmental isolates. Results of pathogenicity experiments for *Vp.* 1474, *Vp.* 1496, and *Vp.* 1513, which were selected on the basis of differences in hemolysin expression and biofilm formation, showed a significant difference in the pathogenicity of the three strains in mice, with a marked distinction regarding the fatality and lethal time, with *Vp.* 1496^tdh-,trh-^ being the most lethal. Considerable differences in the pathogenicity of *V. parahaemolyticus* environmental isolates indicated their strong environmental and host adaptability (Liu et al., 2016), which may be one of the major reasons behind their distribution worldwide (Lovell, 2017).

#### TNSS

TNSS is involved in multiple pathogenic mechanisms during *V. parahaemolyticus* infection. The T3SS1 gene cluster is related to cytotoxic activity and is commonly found in *V. parahaemolyticus*; the T3SS2 gene cluster is mainly found in clinical isolates and is associated with intestinal toxicity (Ham and Orth, 2012). Another secretory system found in *V. parahaemolyticus*, T6SS, is also responsible for the pathogenicity of this species. T6SS is divided into two types, T6SS1 and T6SS2. T6SS1, mainly found in clinical isolates, improves adhesion to hosts (Yu et al., 2012). Recently, a large and broad population study of *V. parahaemolyticus* by Yang and Pei et al. also referred that T6SS was differentiated between sampling groups, which could be involved in environment fitness difference of this species, and it was noteworthy (Yang et al., 2019a).

In our study, the results of *V. parahaemolyticus in vivo* infection showed a significant difference of pathogenicity between the three selected strains, with *Vp.* 1496 being the most pathogenic. Combined with the results of genes annotated to T3SS, there was a lack of difference in T3SS prediction in the three strains, which partially indicated that the pathogenic properties of *Vp.* 1496 might not be developed from the T3SS. Conversely, the marked increase in the number of T6SS-associated protein in *Vp.* 1496 suggests that the pathogenic properties of this strain were most likely enhanced by T6SS-associated mechanisms. Previous literature showed that T6SS might be related to environmental fitness, but not to pathogenicity of foodborne *V. parahaemolyticus* due to its high frequency of distribution (Yang et al., 2019b). However, based on our research, it may be stated that T6SS in foodborne *V. parahaemolyticus* requires further surveillance, for its possible role in *V. parahaemolyticus* infection.

### Antibiotic Resistance of *V. parahaemolyticus*

The other significant biological process in the pathogenicity of *V. parahaemolyticus* lies in cell adhesion and secretion systems. Biofilm formation is the external manifestation of bacterial adhesion ability, which directly affects the antibiotic resistance of bacteria (Carniello et al., 2018). In *Vibrio*, the biofilm formation capacity not only positively reflects colonization stability in the host, but is also closely related to the course of disease after *Vibrio* infection (Khan et al., 2020).

Based on the conclusions drawn from different studies, the drug resistance of *V. parahaemolyticus* is divergent. The antibiotic resistances of strains collected from different locations in the world, and from different sources (clinical isolates and environmental isolates) show a complicated trend (WHO, 2014). However, it must be noted that previous studies on antibiotic resistance of *V. parahaemolyticus* have not been documented as extensively as those for other common foodborne bacteria (Elmahdi et al., 2016).

Based on *de novo* sequencing, the potential antibiotic resistance features of the three tested *V. parahaemolyticus* strains could partially be reflected by ARDB result interpretation, which all showed multiple antibiotic relevant genes annotated to tigecycline (TGC), streptomycin (SM), kanamycin (KAR), CIP, norfloxacin (NFX), and TE. However, some of the antibiotics were not commonly used clinically. Therefore, drug sensitivity test was performed in addition to verify the clinical antibiotic resistance of environmental *V. parahaemolyticus* strains. The antibiotic tests showed different results from those reported in previous studies. The resistance of *V. parahaemolyticus* environmental isolates to AMP was fully confirmed and is consistent with findings of previous studies (Han et al., 2017; Jiang et al., 2019). However, the resistance of *V. parahaemolyticus* environmental isolates to quinolones and cephalosporins, especially to CAZ and CIP, which was confirmed by Lopatek et al. (Lopatek et al., 2018), could not be established by this method. Further, the resistance of *V. parahaemolyticus* environmental isolates to aminoglycosides and TE could not be verified because of the insufficient sample size by this method, while the resistance to TE could be confirmed by ARDB interpretation. The results also suggest that β-lactam antibiotics need to be selected carefully when treating *V. parahaemolyticus* infections, because some of the selected strains were resistant to such antibiotics, including cephalosporin, PRL, KF, and KZ. Finally, the biofilm formation capacity of *V. parahaemolyticus* environmental isolates were weak, and there appeared to be no significant correlation between their biofilm formation and antibiotic resistance.

According to two methods referring to genomics and clinical detection, *V. parahaemolyticus* environmental isolates showed a complicated antibiotic resistance potential. The results suggested that the antibiotic resistance of environmental *V. parahaemolyticus* might be regulated by various mechanisms, and the resistance prediction based on genomics needed to be verified by clinical detection. Similarly, clinical medication when treating *V. parahaemolyticus* infection should be combined with genomic sequencing indication, which need to be studied further.

## Conclusion

Our study analyzed the genetic distribution, genetic elements, and pathogenicity of environmental *V. parahaemolyticus* strains using the WGS method. Our analyses revealed that the difference in pathogenicity of environmental *V. parahaemolyticus* strains resulted from the combination of HGT level, distribution of pathogenic elements, and the nature of the secretory system. Thus, further research on the genome differences between environmental and clinical *V. parahaemolyticus* is necessary for developing a better understanding of these pathogenic bacteria.

## Data Availability Statement

The original contributions presented in the study are publicly available. This data can be found here: NCBI repository, accession number: PRJNA722971 (https://www.ncbi.nlm.nih.gov/bioproject/PRJNA722971).

## Ethics Statement

The animal study was reviewed and approved by Animal Ethics Committee of the Sixth Medical Centre of Chinese PLA General Hospital.

## Author Contributions

LZ conceived this study. JL and KQ designed the research. JL, KQ, and CW performed the experiments. KF and XY analyzed experimental data. JL, KQ, CW, and LZ wrote the paper. JL and KQ contributed equally to this work. All authors contributed to the article and approved the submitted version.

## Funding

This work was supported by the Medical and Health key Project under grant no. 14J004; the Innovation Project of General Hospital under grant no. CX19027; the Innovation Incubation Fund of the Navy General Hospital under grant no. CXPY201822 and CXPY201824; and the Beijing Municipal Natural Science Foundation under grant no. 7204314.

## Conflict of Interest

The authors declare that the research was conducted in the absence of any commercial or financial relationships that could be construed as a potential conflict of interest.
