# Supplementary material for: De Novo Sequencing Provides Insights Into the Pathogenicity of Foodborne Vibrio parahaemolyticus
Source: Front Cell Infect Microbiol. 2021 May 14;11:652957. doi: 10.3389/fcimb.2021.652957 (PMC8162212; doi:10.3389/fcimb.2021.652957)
Supplement: Supplementary file 1 [file DataSheet_1.pdf]

Table S1 | Information of bacterial strains used in this study.

Strain name	Application	Source	remarks
V. parahaemolyticus 1463	Tested strain	Commercial seafood isolated from Anhui province	Isolation date: 1st Jun, 2016
V. parahaemolyticus 1464	Tested strain	Commercial seafood isolated from Anhui province	Isolation date: 1st Jun, 2016
V. parahaemolyticus 1465	Tested strain	Commercial seafood isolated from Anhui province	Isolation date: 1st Jun, 2016
V. parahaemolyticus 1466	Tested strain	Commercial seafood isolated from Anhui province	Isolation date: 1st Jun, 2016
V. parahaemolyticus 1467	Tested strain	Commercial seafood isolated from Anhui province	Isolation date: 1st Jun, 2016
V. parahaemolyticus 1468	Tested strain	Commercial seafood isolated from Anhui province	Isolation date: 1st Jun, 2016
V. parahaemolyticus 1469	Tested strain	Commercial seafood isolated from Anhui province	Isolation date: 1st Jun, 2016
V. parahaemolyticus 1470	Tested strain	Commercial seafood isolated from Anhui province	Isolation date: 1st Jun, 2016
V. parahaemolyticus 1471	Tested strain	Commercial seafood isolated from Anhui province	Isolation date: 1st Jun, 2016
V. parahaemolyticus 1472	Tested strain	Commercial seafood isolated from Anhui province	Isolation date: 1st Jun, 2016
V. parahaemolyticus 1473	Tested strain	Commercial seafood isolated from Anhui province	Isolation date: 1st Jun, 2016
V. parahaemolyticus 1474	Tested strain	Commercial seafood isolated from Anhui province	Isolation date: 1st Jun, 2016
V. parahaemolyticus 1475	Tested strain	Commercial seafood isolated from Anhui province	Isolation date: 1st Jun, 2016
V. parahaemolyticus 1476	Tested strain	Commercial seafood isolated from Anhui province	Isolation date: 1st Jun, 2016
V. parahaemolyticus 1477	Tested strain	Commercial seafood isolated from Anhui province	Isolation date: 1st Jun, 2016
V. parahaemolyticus 1478	Tested strain	Commercial seafood isolated from Anhui province	Isolation date: 1st Jun, 2016
V. parahaemolyticus 1479	Tested strain	Commercial seafood isolated from Anhui province	Isolation date: 1st Jun, 2016
V. parahaemolyticus 1480	Tested strain	Commercial seafood isolated from Anhui province	Isolation date: 1st Jun, 2016
V. parahaemolyticus 1481	Tested strain	Commercial seafood isolated from Anhui province	Isolation date: 1st Jun, 2016
V. parahaemolyticus 1482	Tested strain	Commercial seafood isolated from Anhui province	Isolation date: 1st Jun, 2016
V. parahaemolyticus 1483	Tested strain	Commercial seafood isolated from Anhui province	Isolation date: 1st Jun, 2016
V. parahaemolyticus 1484	Tested strain	Commercial seafood isolated from Shanxi province	Isolation date: 1st Jun, 2016

Strain name	Application	Source	remarks
V. parahaemolyticus 1485	Tested strain	Commercial seafood isolated from Shanxi province	Isolation date: 1st Jun, 2016
V. parahaemolyticus 1486	Tested strain	Commercial seafood isolated from Shanxi province	Isolation date: 1st Jun, 2016
V. parahaemolyticus 1487	Tested strain	Commercial seafood isolated from Shanxi province	Isolation date: 1st Jun, 2016
V. parahaemolyticus 1488	Tested strain	Commercial seafood isolated from Shanxi province	Isolation date: 1st Jun, 2016
V. parahaemolyticus 1489	Tested strain	Commercial seafood isolated from Shanxi province	Isolation date: 1st Jun, 2016
V. parahaemolyticus 1490	Tested strain	Commercial seafood isolated from Shanxi province	Isolation date: 1st Jun, 2016
V. parahaemolyticus 1491	Tested strain	Commercial seafood isolated from Shanxi province	Isolation date: 1st Jun, 2016
V. parahaemolyticus 1492	Tested strain	Commercial seafood isolated from Shanxi province	Isolation date: 1st Jun, 2016
V. parahaemolyticus 1493	Tested strain	Commercial seafood isolated from Shanxi province	Isolation date: 1st Jun, 2016
V. parahaemolyticus 1494	Tested strain	Commercial seafood isolated from Shanxi province	Isolation date: 1st Jun, 2016
V. parahaemolyticus 1495	Tested strain	Commercial seafood isolated from Shanxi province	Isolation date: 1st Jun, 2016
V. parahaemolyticus 1496	Tested strain	Commercial seafood isolated from Shanxi province	Isolation date: 1st Jun, 2016
V. parahaemolyticus 1497	Tested strain	Commercial seafood isolated from Fujian province	Isolation date: 1st Jun, 2016
V. parahaemolyticus 1498	Tested strain	Commercial seafood isolated from Fujian province	Isolation date: 1st Jun, 2016
V. parahaemolyticus 1499	Tested strain	Commercial seafood isolated from Fujian province	Isolation date: 1st Jun, 2016
V. parahaemolyticus 1500	Tested strain	Commercial seafood isolated from Fujian province	Isolation date: 1st Jun, 2016
V. parahaemolyticus 1501	Tested strain	Commercial seafood isolated from Fujian province	Isolation date: 1st Jun, 2016
V. parahaemolyticus 1502	Tested strain	Commercial seafood isolated from Fujian province	Isolation date: 1st Jun, 2016
V. parahaemolyticus 1503	Tested strain	Commercial seafood isolated from Fujian province	Isolation date: 1st Jun, 2016
V. parahaemolyticus 1504	Tested strain	Commercial seafood isolated from Fujian province	Isolation date: 1st Jun, 2016
V. parahaemolyticus 1505	Tested strain	Commercial seafood isolated from Fujian province	Isolation date: 1st Jun, 2016
V. parahaemolyticus 1506	Tested strain	Commercial seafood isolated from Fujian province	Isolation date: 1st Jun, 2016
V. parahaemolyticus 1507	Tested strain	Commercial seafood isolated from Fujian province	Isolation date: 1st Jun, 2016
V. parahaemolyticus 1508	Tested strain	Commercial seafood isolated from Fujian province	Isolation date: 1st Jun, 2016

Strain name	Application	Source	remarks
V. parahaemolyticus 1509	Tested strain	Commercial seafood isolated from Fujian province	Isolation date: 1st Jun, 2016
V. parahaemolyticus 1510	Tested strain	Commercial seafood isolated from Fujian province	Isolation date: 1st Jun, 2016
V. parahaemolyticus 1511	Tested strain	Commercial seafood isolated from Fujian province	Isolation date: 1st Jun, 2016
V. parahaemolyticus 1512	Tested strain	Commercial seafood isolated from Fujian province	Isolation date: 1st Jun, 2016
V. parahaemolyticus 1513	Tested strain	Commercial seafood isolated from Fujian province	Isolation date: 1st Jun, 2016
V. parahaemolyticus 1514	Tested strain	Commercial seafood isolated from Fujian province	Isolation date: 1st Jun, 2016
V. parahaemolyticus 1515	Tested strain	Commercial seafood isolated from Fujian province	Isolation date: 1st Jun, 2016
V. parahaemolyticus 1516	Tested strain	Commercial seafood isolated from Fujian province	Isolation date: 1st Jun, 2016
V. parahaemolyticus 1517	Tested strain	Commercial seafood isolated from Fujian province	Isolation date: 1st Jun, 2016
V. parahaemolyticus 1518	Tested strain	Commercial seafood isolated from Fujian province	Isolation date: 1st Jun, 2016
V. parahaemolyticus 1519	Tested strain	Commercial seafood isolated from Fujian province	Isolation date: 1st Jun, 2016
V. parahaemolyticus 1520	Tested strain	Commercial seafood isolated from Fujian province	Isolation date: 1st Jun, 2016
V. parahaemolyticus 1521	Tested strain	Commercial seafood isolated from Fujian province	Isolation date: 1st Jun, 2016
V. parahaemolyticus 1522	Tested strain	Commercial seafood isolated from Fujian province	Isolation date: 1st Jun, 2016
V. parahaemolyticus 1523	Tested strain	Commercial seafood isolated from Fujian province	Isolation date: 1st Jun, 2016
V. parahaemolyticus 1524	Tested strain	Commercial seafood isolated from Fujian province	Isolation date: 1st Jun, 2016
V. parahaemolyticus 1525	Tested strain	Commercial seafood isolated from Fujian province	Isolation date: 1st Jun, 2016
V. parahaemolyticus 1526	Tested strain	Commercial seafood isolated from Fujian province	Isolation date: 1st Jun, 2016
V. parahaemolyticus 1527	Tested strain	Commercial seafood isolated from Fujian province	Isolation date: 1st Jun, 2016
V. parahaemolyticus 1528	Tested strain	Commercial seafood isolated from Fujian province	Isolation date: 1st Jun, 2016
V. parahaemolyticus 4213	Tested strain	Commercial seafood isolated from Fujian province	Isolation date: 12th Mar, 2016
V. parahaemolyticus 4215	Tested strain	Commercial seafood isolated from Fujian province	Isolation date: 12th Mar, 2016
V. parahaemolyticus 11577	Tested strain	Commercial seafood isolated from Fujian province	Isolation date: 25th Aug, 2015
V. probioticus LMG 20362T	Reference strain	*Litopenaeus vannamei*	GenBank accession No. AJ345063.1

Strain name	Application	Source	remarks
V. rotiferianus LMG 21460T	Reference strain	*Brachionus plicatilis*	GenBank accession No. AJ316187.1
V. proteolyticus ATCC 15338T	Reference strain	*Limnoria tripunctata*	GenBank accession No. X74723.1
V. parahaemolyticus ATCC 17802T	Reference strain	human patients of Shirasu food poisoning	GenBank accession No. NR_041838.1
V. natriegens ATCC 14048T	Reference strain	Salt marsh mud	GenBank accession No. X74714.1
V. harveyi NCIMB 1280T	Reference strain	*Talorchestria* sp.	GenBank accession No. AY750575.1
V. campbellii ATCC 25920T	Reference strain	seawater	GenBank accession No. X56575.1
V. alginolyticus ATCC 17749T	Reference strain	horse mackerel causing food poisoning	GenBank accession No. X56576.1
E. coli ATCC 25922	Reference strain	clinical isolate	CLSI M45-A2 recommended

Table S2 | List of primers targeted on hemolysins of *V. parahaemolyticus*.

Targeted gene	Primer description	Primer sequence	GenBank accession
tdh	forward	5’-GTAAAGGTCTCTGACTTTTGGAC-3’	No. M10069.1
	reverse	5’-TGGAATAGAACCTTCATCTTCACC-3’	
trh	forward	5’-TTGGCTTCGATATTTTCAGTATCT-3’	No. KP836472.1
	reverse	5’ -CATAACAACATATGCCCATTTCCG-3’	
tlh	forward	5’ -AAAGCGGATTATGCAGAAGCACTG-3’	No. AY289609.1
	reverse	5’ -GCTACTTTCTAGCATTTTCTCTGC-3’	

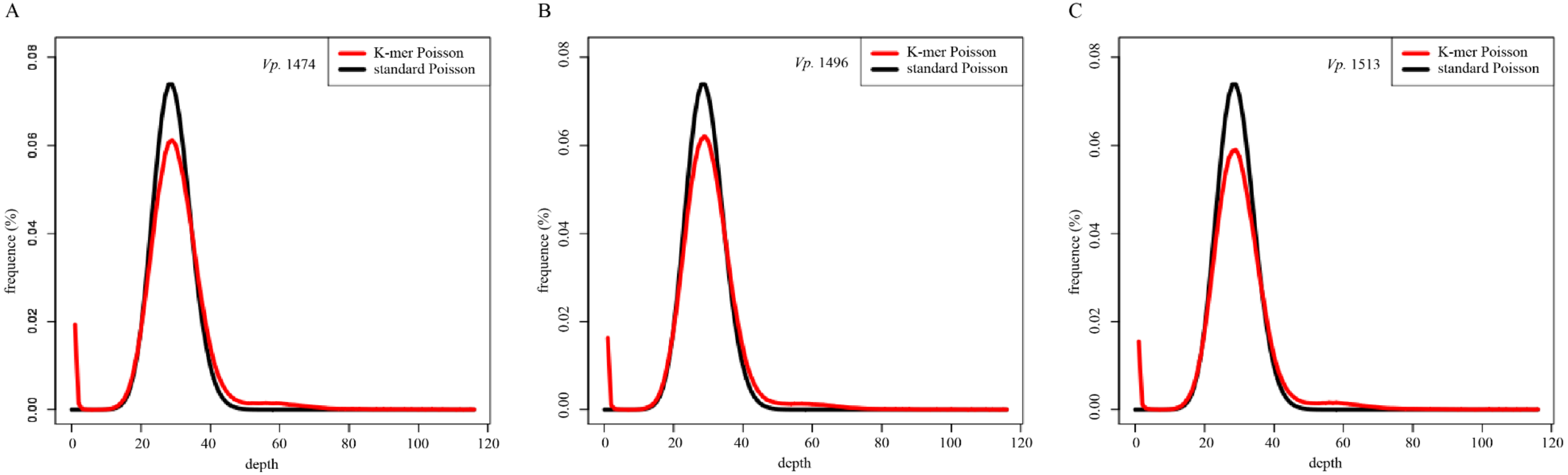

D

Statistics of 15-K-mer analysis							
Description / *Vp.* strain	K-mer length	K-mer number	K-mer depth	Original genome size (Mb)	Revised genome size (Mb)	Heterozygous rate (%)	Repeat rate (%)
Vp. 1474	15	266388916	49.87	5.34	5.25	0.00	10.40
Vp. 1496	15	267011539	52.18	5.12	5.03	0.01	11.07
Vp. 1513	15	267231880	47.55	5.62	5.53	0.01	14.52

Figure S1 | K-mer frequency distribution of *V. parahaemolyticus*.

(A) 15-K-mer analysis of strain *Vp*. 1474. **(B)** 15-K-mer analysis of strain *Vp*. 1496. **(C)** 15-K-mer analysis of strain *Vp*. 1513. **(D)** Statistics of 15-K-mer analysis. Red curve: K-mer poisson; Black curve: standard poisson.

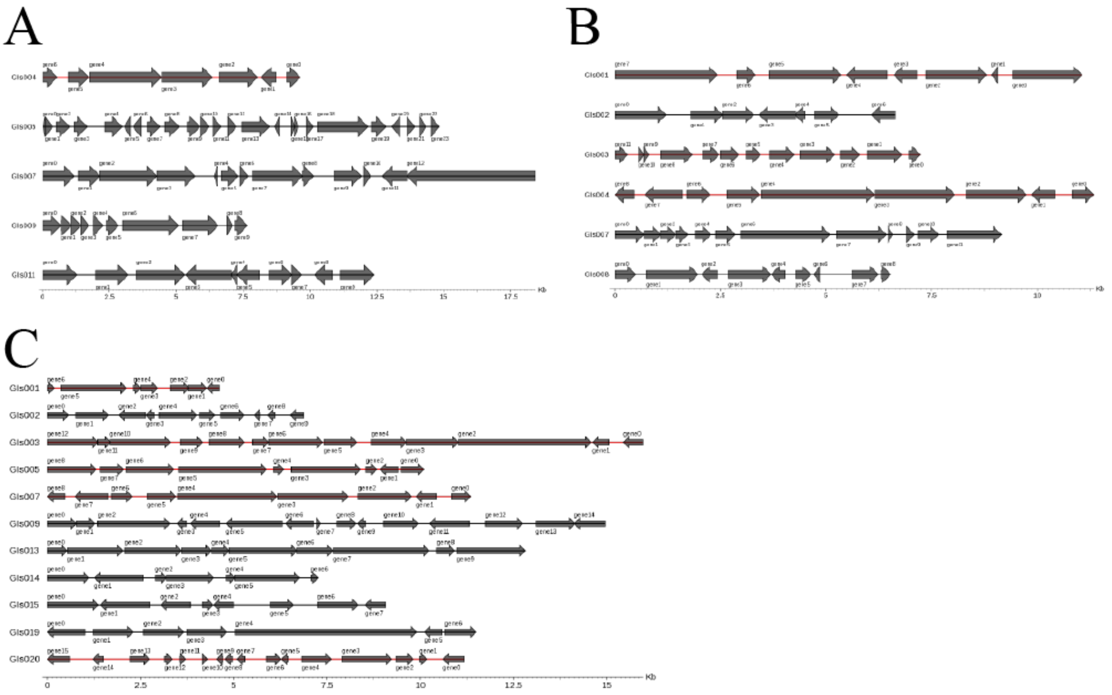

D

Characteristics of the predicted GIs

Vp. strain \ Description	GI ID	Sequence location	Start sequence base	End sequence base	GI length	Gene number
Vp. 1474	GIs001	scaffold 1	118500	140226	21727	22
	GIs002	scaffold 1	142624	209106	66483	55
	GIs003	scaffold 1	212001	228475	16475	15
	GIs004	scaffold 1	1120541	1130170	9630	7
	GIs005	scaffold 2	635505	650216	14712	24
	GIs006	scaffold 3	24717	58615	33899	32
	GIs007	scaffold 5	53785	67402	13618	13
	GIs008	scaffold 6	60921	77576	16656	14
	GIs009	scaffold 8	117102	124759	7658	10
	GIs010	scaffold 8	163792	179603	15812	17
	GIs011	scaffold 9	9148	21538	12391	10
Vp. 1496	GIs001	scaffold 1	374021	385074	11054	8
	GIs002	scaffold 1	828460	835090	6631	7
	GIs003	scaffold 2	454379	461606	7228	12
	GIs004	scaffold 2	1152194	1163518	11325	9
	GIs005	scaffold 3	147666	174950	27285	23
	GIs006	scaffold 3	404608	436696	32089	30
	GIs007	scaffold 5	123450	132592	9143	12
	GIs008	scaffold 8	24174	30697	6524	9

Vp. strain \ Description	GI ID	Sequence location	Start sequence base	End sequence base	GI length	Gene number
Vp. 1513	GIs001	scaffold 11	10206	14816	4611	7
	GIs002	scaffold 13	59893	66762	6870	10
	GIs003	scaffold 1	464142	478762	14621	13
	GIs004	scaffold 1	483187	509388	26202	25
	GIs005	scaffold 1	724061	734151	10091	9
	GIs006	scaffold 1	982260	998858	16599	14
	GIs007	scaffold 1	1062597	1073943	11347	9
	GIs008	scaffold 2	41465	64275	22811	25
	GIs009	scaffold 2	72281	85657	13377	15
	GIs010	scaffold 2	610991	627696	16706	14
	GIs011	scaffold 3	696040	726594	30555	30
	GIs012	scaffold 3	743994	765306	21313	18
	GIs013	scaffold 3	869534	882343	12810	10
	GIs014	scaffold 4	164020	171280	7261	7
	GIs015	scaffold 4	367452	376510	9059	8
	GIs016	scaffold 4	398301	415111	16811	19
	GIs017	scaffold 5	430138	462061	31924	29
	GIs018	scaffold 7	71621	93337	21717	24
	GIs019	scaffold 8	6604	18097	11494	7
	GIs020	scaffold 9	28720	39888	11169	16

Figure S2 | Characteristics and gene distribution of predicted GIs of *V. parahaemolyticus*.

(A) Gene distribution in short GIs (<15 Kb) of strain *Vp*. 1474. **(B)** Gene distribution in short GIs (<15 Kb) of strain *Vp*. 1496. **(C)** Gene distribution in short GIs (<15 Kb) of strain *Vp*. 1513. **(D)** Statistical data of GIs prediction for the three strains.
